# ﻿A widespread new genus of Baetidae (Baetidae, Ephemeroptera) from Southeast Asia

**DOI:** 10.3897/zookeys.1135.93800

**Published:** 2022-12-12

**Authors:** Thomas Kaltenbach, Nikita J. Kluge, Jean-Luc Gattolliat

**Affiliations:** 1 Museum of Zoology, Palais de Rumine, Place Riponne 6, CH-1005 Lausanne, Switzerland Museum of Zoology, Palais de Rumine Lausanne Switzerland; 2 University of Lausanne (UNIL), Department of Ecology and Evolution, CH-1015 Lausanne, Switzerland University of Lausanne (UNIL) Lausanne Switzerland; 3 Department of Entomology, Biological Faculty, Saint-Petersburg State University, Universitetskaya nab., 7/9, Saint Petersburg, 199034, Russia Saint Petersburg State University Saint Petersburg Russia

**Keywords:** Accessory gills, COI, Indonesia, integrated taxonomy, Malaysia, Philippines

## Abstract

A reinvestigation of type and other material of *Baetisjavanicus* Ulmer, 1913 and *Baetissabahensis* Müller-Liebenau, 1984, together with new material from Southeast Asia revealed a new genus, *Branchiobaetis***gen. nov.** The above species are formally assigned to the new genus *Branchiobaetis***gen. nov.** It is characterized by the presence of accessory gills ventrally near fore coxa and at the base of maxillae, a peculiar folding of the gonostyli developing under the cuticle of last instar male larvae, together with a unique combination of other larval characters. Besides the two formerly described species, five new species are identified using a combination of morphology and molecular characters (COI, Kimura 2-parameter distances), four species from Sumatra and one from the Philippines. They are described and illustrated at the larval stage. Additionally, a complementary description of larva and adult stages of the generic type species *B.javanicus***comb. nov.** as well as the first description of the eggs are provided. Furthermore, new reports of *B.javanicus***comb. nov.** and *B.sabahensis***comb. nov.** are indicated. The distribution of *Branchiobaetis***gen. nov.** includes the Indonesian Sunda Islands, Borneo, and the Philippines. A key to the larval stage of all species is provided.

## ﻿Introduction

Baetidae are the family with the highest species diversity among mayflies on species and generic level. They comprise ca. 1,100 species in 114 genera (Kluge 2022), which is close to one third of all mayfly species and approximately one quarter of all mayfly genera worldwide. They have a cosmopolitan distribution except New Zealand (Gattolliat and Nieto 2009). Investigations of the molecular phylogeny of the Order Ephemeroptera revealed the relatively basal position of the family in Ephemeroptera phylogeny (Ogden et al. 2019).

The different realms were not equally studied in the past, and especially the Baetidae of the megadiverse Southeast Asia and New Guinea are still poorly known, despite substantial progress in the last decade with the establishment of several genera and many new species (e.g., Kluge and Novikova 2011, 2017; Gattolliat 2012; Kluge 2012, 2016; Kaltenbach and Gattolliat 2018, 2019, 2021; Kaltenbach et al. 2020a, b, 2021; Kluge 2020; Kluge and Suttinun 2020; Kluge et al. 2020; Suttinun et al. 2020).

Here, we describe a new genus of Baetidae with a wide distribution across Southeast Asia. It includes two known species, formerly described in the genus *Baetis* Leach, 1815, and five new species from Indonesia (Sumatra) and the Philippines. The new genus is easily distinguished from all other genera by the presence of accessory gills at the base of maxillae and between fore coxa and prosternum, a peculiar folding of the gonostyli developing under the cuticle of male last instar larvae, plus a unique combination of other larval characters.

Indonesia is an immense archipelago of more than 18.000 islands extending over a huge area from 95°E to 141°E and from 6°N to 11°S. It is one of the most biologically rich countries in the world. The high levels of species richness and endemism are mainly attributable to a complex geological history, which brought together two different biological realms (Oriental and Australasian realms), separated by a transitional region (Wallacea) (Hall 2010; Kingston 2010). The main islands are Sumatra, Java, Borneo (partly, Kalimantan Province), Sulawesi and New Guinea (partly, provinces West Papua and Papua). Borneo, Sumatra, Java, and the Malay Peninsula are forming the Sundaland Biodiversity Hotspot (Quek 2010), influenced by a dynamic and highly complex geophysical history including changing climates, fluctuating sea levels, volcanism, and orogenic activity with subsequent erosion (Quek 2010).

Similarly, the Philippines are a complex archipelago with more than 7100 islands, spanning the Asian-Australian faunal zone interface directly at the Wallace Line. The Huxley Line is dividing the country into Palawan and associated islands, the presumed former land-bridge to northern Borneo, and the truly oceanic portions of the Philippines. It possesses an extraordinary biodiversity, presumably supported by ancient land mass movements, environmental gradients along steep volcanic slopes and alterations of connectivity between neighbouring islands induced by changing sea levels (Brown and Diesmos 2010).

Taking into account the extreme diversity in Southeast Asia, the rather poor collection activities in the past, with many still unexplored regions, and the obvious richness of Baetidae in this region, we have to expect further new genera and many more species with further collections in the future.

## ﻿Materials and methods

The larvae were collected by kick-sampling and preserved in 70–96% ethanol. For some of the new species, ecological data were gathered by Morgan Gueuning (University of Lausanne, **UNIL**) during his own studies (Gueuning et al. 2017).

Subimagos were reared by one of us (NK) from mature larvae in cages placed in the stream. Subsequently, female imago was reared from subimago placed in a container with wet air, but without water. Imagos and subimagos were individually associated with larval and subimaginal exuviae.

The dissection of larvae was done in Cellosolve (2-Ethoxyethanol) with subsequent mounting on slides with Euparal liquid, using an Olympus SZX7 stereomicroscope. Alternatively, dissection was done in alcohol with subsequent mounting on slides with Canada balsam, using a stereomicroscope MSP 2; and examination with microscope Leica DM 1000.

The DNA of part of the specimens was extracted using non-destructive methods allowing subsequent morphological analysis (see Vuataz et al. 2011 for details). We amplified a 658 bp fragment of the mitochondrial gene cytochrome oxidase subunit 1 (COI) using the primers LCO 1490 and HCO 2198 (Folmer et al. 1994, see Kaltenbach and Gattolliat 2020 for details). Sequencing was done with Sanger’s method (Sanger et al. 1977). The genetic variability between specimens was estimated using Kimura-2-parameter distances (K2P, Kimura 1980), calculated with the program MEGA 11 (Tamura et al. 2021, http://www.megasoftware.net). COI sequencing was done for species delimitation only. To compare COI divergence to our morphological identifications, we applied the single-locus species delimitation method ASAP (Assemble Species by Automatic Partitioning; Puillandre et al. 2020) to our COI data set. We used the ASAP webserver available at https://bioinfo.mnhn.fr/abi/public/asap/asapweb.html, computing the genetic distances under the Kimura 2-parameter substitution model (Kimura 1980) with all other settings set to default. The ASAP method, which is an improvement of the widely used ABGD (Automatic Barcode Gap Discovery; Puillandre et al. 2012) approach, has the advantage of providing a score that designates the most likely number of hypothetical species. Further, a phylogenetic reconstruction with Maximum Likelihood (Bootstrap, 1000 replications) was done with MEGA 11 (Suppl. material 1). HKY+G+I was the best-fit substitution model.

The GenBank accession numbers are given in Table 1; nomenclature of gene sequences follows Chakrabarty et al. (2013).

**Table 1.** Sequenced specimens of *Branchiobaetis* gen. nov.

Species	Locality	Specimen voucher	GenBank #	GenSeq
		catalogue #	(COI)	Nomenclature
B.cf.javanicus comb. nov.	Indonesia: Sumbawa	GBIFCH00980895	OP279184	genseq-4 COI
		GBIFCH00980896	OP279185	genseq-4 COI
	Indonesia: Bali	GBIFCH00980902	OP279186	genseq-4 COI
	Indonesia: Sumatra	GBIFCH00980893	OP279187	genseq-4 COI
		GBIFCH00980894	OP279188	genseq-4 COI
*B.aduncus* sp. nov.	Indonesia: Sumatra	GBIFCH00422219	OP279189	genseq-1 COI
*B.hamatus* sp. nov.	Indonesia: Sumatra	GBIFCH00422261	OP279192	genseq-1 COI
		GBIFCH01116020	OP279190	genseq-2 COI
		GBIFCH01115975	OP279191	genseq-2 COI
*B.joachimi* sp. nov.	Indonesia: Sumatra	GBIFCH00422238	OP279195	genseq-2 COI
		GBIFCH00422259	OP279194	genseq-2 COI
		GBIFCH00422248	OP279196	genseq-2 COI
		GBIFCH00980903	OP279193	genseq-2 COI
		GBIFCH00980898	OP279197	genseq-4 COI
*B.minangkabau* sp. nov.	Indonesia: Sumatra	GBIFCH00422480	OP279200	genseq-2 COI
		GBIFCH00406299	OP279198	genseq-2 COI
		GBIFCH00980904	OP279199	genseq-2 COI
*B.jhoanae* sp. nov.	Philippines	GBIFCH00980901	OP279201	genseq-2 COI

Drawings were made using an Olympus BX43 microscope. To facilitate the determination of species and the comparison of important structures, we partly used a combination of dorsal and ventral aspects in one drawing. Explanations are given in Kaltenbach et al. (2020a: fig. 1).

**Figure 1.**
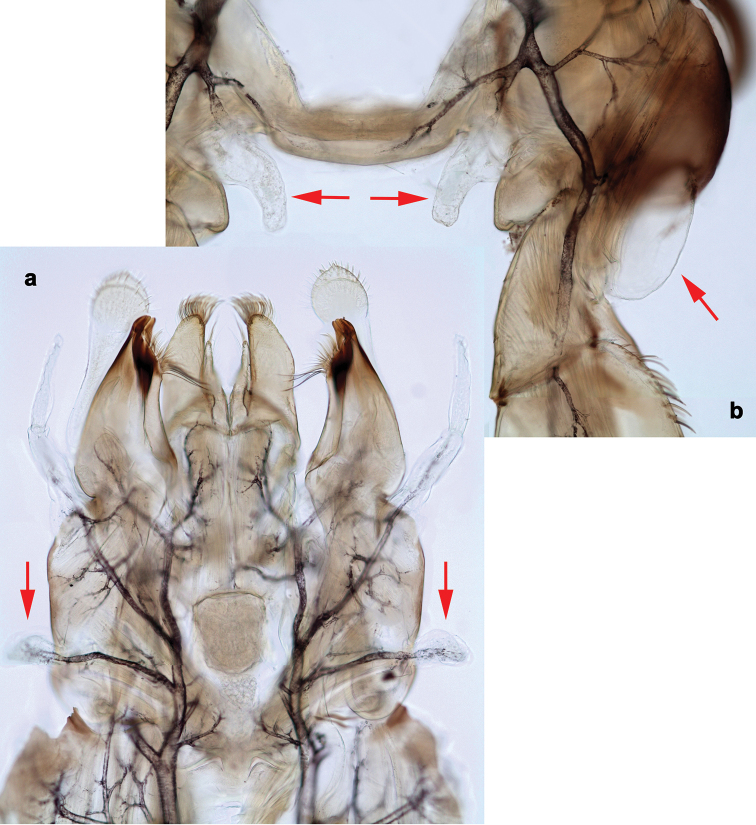
*Branchiobaetisjavanicus* comb. nov., larva **a** maxillae and labium, dorsal view **b** prosternum and bases of forelegs, front view.

Photographs of larvae were taken using a Canon EOS 6D camera and processed with the programs Adobe Photoshop Lightroom (http://www.adobe.com) and Helicon Focus v. 5.3 (http://www.heliconsoft.com). Images of larval parts were taken with a DMC 4500 camera on a Leica M205C stereomicroscope and an Olympus SC 50 camera on an Olympus BX51 microscope, processed with the program Olympus Stream Basic.

Photographs were subsequently enhanced with Adobe Photoshop Elements 13.

The distribution maps were generated with the program SimpleMappr (https://simplemappr.net, Shorthouse 2010). Google Earth (http://www.google.com/earth/download/ge/) was used to attribute approximate GPS coordinates to elder sample locations (Table 2).

**Table 2.** GPS coordinates of locations of *Branchiobaetis* gen. nov. (LT: locus typicus).

Species	Country	Location	Coordinates	LT
*B.javanicus* comb. nov.	Indonesia	Java: Bogor	06°35'32"S, 106°48'00"E	
			06°39'29"S, 106°44'55"E	
			06°30'48"S, 107°00'03"E	
		Java: Dieng Plateau	07°12'54"S, 109°53'58"E	
		Java: Gunung Gede	06°47'16"S, 106°58'55"E	x
		Java: Gunung Ungaran	07°11'01"S, 110°20'54"E	
		Java: Malang Batu	07°54'52"S, 112°35'05"E	
		Java: Ranu Bedali	07°57'03"S, 113°16'16"E	
		Java: Sarangan	07°39'50"S, 111°12'14"E	
		Java: Tjibodas (Cibodas)	06°44'29"S, 107°00'27"E	
		Java: Tjisarua (Cisarua)	06°39'30"S, 106°28'03"E	
		Lombok	08°25'32"S, 116°23'45"E	
B.cf.javanicus comb. nov.		Bali	08°29'59"S, 115°14'35"E	
			08°31'10"S, 115°15'18"E	
		Flores	08°42'55"S, 122°04'24"E	
		Sumatra	00°54'40"S, 100°28'23"E	
		Sumatra: Ranau	04°51'04"S, 103°56'15"E	
		Sumatra: Tjurup	03°27'45"S, 102°30'18"E	
		Sumba	09°38'37"S, 119°40'56"E	
		Sumbawa	08°35'52"S, 117°16'41"E	
*B.sabahensis* comb. nov.	Malaysia	Sabah (Borneo)	05°51'48"N,116°15'37"E	x
			05°57'13"N,116°39'50"E	
			05°59'10"N,116°34'42"E	
B.cf.sabahensis comb. nov.	Indonesia	East Kalimantan (Borneo)	02°59'22"N,116°30'46"E	
			02°59'20"N,116°33'11"E	
			03°00'57"N,116°32'16"E	
*B.aduncus* sp. nov.	Indonesia	Sumatra: volcano Singgalang	00°23'03"S, 100°21'24"E	x
	Indonesia	Sumatra: Aceh	03°58'36"N,97°15'17"E	
	Indonesia	Sumatra: Talang	00°52'52"S, 100°37'23"E	
*B.hamatus* sp. nov.	Indonesia	Sumatra: volcano Talamau	00°02'59"N,100°00'01"E	x
		Sumatra: volcano Singgalang	00°19'57"S, 100°19'19"E	
*B.joachimi* sp. nov.	Indonesia	Sumatra: volcano Marapi	00°21'33"S, 100°30'42"E	x
		Sumatra: volcano Sago	00°22'33"S, 100°39'33"E	
			00°22'20"S, 100°41'45"E	
			00°20'37"S, 100°41'02"E	
			00°18'01"S, 100°40'08"E	
		Sumatra: volcano Singgalang	00°24'07"S, 100°16'44"E	
			00°23'33"S, 100°16'34"E	
			00°22'50"S, 100°17'39"E	
		Sumatra: above Padang	00°56'44"S, 100°32'44"E	
*B.minangkabau* sp. nov.	Indonesia	Sumatra: volcano Talamau	00°02'15"S, 99°59'24"E	x
		Sumatra: Sawahlunto	00°35'52"S, 100°43'02"E	
*B.jhoanae* sp. nov.	Philippines	Luzon	12°44'N, 124°05E	x
		Cebu	10°24'56"N,123°49'02"E	
			10°20'48"N,123°51'57"E	

The dichotomous key was elaborated with the support of the program DKey v.1.3.0 (http://drawwing.org/dkey, Tofilski 2018).

The terminology follows Hubbard (1995; legs orientation) and Kluge (2004).

### ﻿Abbreviations of depositories

**AdMU** Ateneo de Manila University, Quezon City (Philippines);

**MZB**Museum Zoologicum Bogoriense (Indonesia);

**MZL**Musée de Zoologie Lausanne (Switzerland);

**PNM**Museum of Natural History of the Philippine National Museum, Manila (Philippines);

**SPbU** Saint-Petersburg State University (Russia);

**ZMH**Zoologisches Museum Hamburg (Germany).

## ﻿Results

### ﻿ Branchiobaetis gen. nov.

Taxon classificationAnimaliaEphemeropteraBaetidae

F12341AD-0D1D-5F9D-980E-A9789EB1EC84

https://zoobank.org/13E7F863-CCA5-4EAD-87F2-423286D897B7

Figs 1 , 2 , 3 , 4 , 5 , 6 , 7 , 8 , 9 , 10 , 11 , 12 , 13 , 14 , 15 , 16 , 17 , 18 , 19 , 20 , 21 , 22 , 23 , 24 , 25 , 26 , 27 , 28 , 29

#### Type species.

*Branchiobaetisjavanicus* (Ulmer, 1913), comb. nov., by present designation.

#### Species included in *Branchiobaetis* gen. nov.

New combinations

1. *Branchiobaetisjavanicus* (Ulmer, 1913), comb. nov.

2. *Branchiobaetissabahensis* (Müller-Liebenau, 1984), comb. nov.

New species from Sumatra

3. *Branchiobaetisaduncus* sp. nov.

4. *Branchiobaetishamatus* sp. nov.

5. *Branchiobaetisjoachimi* sp. nov.

6. *Branchiobaetisminangkabau* sp. nov.

New species from the Philippines

7. *Branchiobaetisjhoanae* sp. nov.

#### Diagnosis.

**Larva.** This new genus is distinguished by a combination of the following characters: A) body elongate and slender (Figs 24a, 25b); B) antennal scape distally with short, stout setae (Fig. 15i); flagellum with basal segments parallel sided and thereafter inclined, giving the impression of a spiral arrangement (Fig. 5a); C) labrum subrectangular, dorsally with a pair of long, simple submedian setae and a submarginal arc of long, simple setae (Fig. 14a); D) right mandible with blade-like incisor, prostheca stick-like with distolateral dentation (Fig. 6b); E) left mandible with blade-like incisor, prostheca robust, distally with denticles and comb-shape structure (Fig. 6a); F) maxillary palp 2-segmented, apex of segment II pointed; with accessory gill outside laterally between stipes and cardo (Figs 1a, 18h–j); G) labium with glossae basally broad, narrowing towards apex, shorter than paraglossae; paraglossae laterally slightly undulated, distally truncate and slightly bent inwards; labial palp with small to medium protuberance at segment II (Fig. 14j); H) femora with stout setae both on anterior and posterior side, dorsal margin with row of medium to long, spine-like setae and straight row of medium, fine setae (Figs 3a, b, 15a, d); claw robust, pointed, with one row of denticles and usually a long, subapical seta (Figs 7k, 15e); femoral patch reduced on fore and middle legs, well developed on hind legs (Fig. 7d–i); I) finger-like accessory gill ventrally between coxa and prosternum (Fig. 1b); J) hind protoptera present, well developed; K) paraproct with spines at posterior margin (Fig. 15h); L) seven pairs of tergalii (abdominal gills) on segments I–VII, anal margin with alternate short and long, fine setae (Fig. 15g); M) subimaginal gonostyli developing under cuticle of last instar larvae folded in the following way: segment II sharply bent towards middle, last segment sharply bent laterally (Figs 4a–d, 10a, b).

**Imago.** Forewing with double intercalary veins longer than the distance between corresponding longitudinal vein; pterostigma with numerous cross veins (Fig. 9a, c). Hind wing with three longitudinal veins and well developed triangular costal projection (Fig. 9d, e). Imaginal gonostyli: segment I of gonostylus with projected blunt angle proximad of its middle; segment III short and triangular (Fig. 10d, e). Sterno-styligeral muscle present and thin (Fig. 10f).

The imago is known for a single species (*B.javanicus* comb. nov.). Therefore, it is unclear, which of its characters are species-specific and which can be considered as diagnostic for the new genus. The structure of hind wing and the presence of a thin sterno-styligeral muscle are also revealed for *B.sabahensis* comb. nov., based on details developing in last instar larvae.

#### Etymology.

*Branchiobaetis* is a combination of *Branchio*-, in reference to the Latin word for gills and the accessory gills of the larvae, and *baetis*, to highlight the similarities with the genus *Baetis*. The gender is masculine.

#### Description.

**Larva.**

**Head. *Antenna***. Bases of antennae not close to each other, without carina between them. Scape at least distally (and often outside laterally) with short, stout, apically rounded setae (Fig. 15i); flagellum with basal segments parallel sided and thereafter inclined, giving the impression of a spiral arrangement (Fig. 5a).

***Labrum*** (Fig. 14a). Subrectangular, wider than long. Distal margin with medial emargination and a small process. Dorsally with a pair of long, simple, submedian setae and on each side a submarginal arc of long, stout, simple setae; surface scattered with medium, simple setae. Ventrally with lateral row of medium, simple setae, anterolaterally with long, feathered setae on margin and medially with long, bifid, pectinate setae on margin, several small, stout setae near anterolateral and sometimes also lateral margin.

***Right mandible*** (Figs 6b, 14b–d, 22b). Incisor and kinetodontium almost fused, incisor with denticles, outer denticle blade-like, kinetodontium with denticles; inner margin of innermost denticle of kinetodontium with row of thin setae; prostheca stick-like, distolaterally denticulate; apex of mola with tuft of feathered setae. Basal half with fine, simple setae scattered over dorsal surface.

***Left mandible*** (Figs 6a, 18e, f). Incisor and kinetodontium fused, incisor with denticles, outer denticle blade-like, kinetodontium with denticles; prostheca robust, distally denticulate and with comb-shape structure; apex of mola without tuft of setae. Basal half with fine, simple setae scattered over dorsal surface.

Incisors of both mandibles are quickly worn after the larva started feeding and become much shorter than in fresh, unused mandibles. The real shape of unused mandibles can be seen during development inside the actual mandible (Figs 6a, b, 20b, d).

***Maxilla*** (Figs 1a, 18h–j). Apically with three stout canines and three denti-setae; distal denti-seta tooth-like, other denti-setae slender, bifid, and pectinate; maxillary palp with two segments, apex strongly pointed. Small accessory gill located on outer side of the articulation between stipes and cardo.

***Hypopharynx*** (Fig. 14h). Apex with compact tuft of long, dense setae-like processes.

***Labium*** (Fig. 14j). Glossae basally broad, narrowing towards apex, shorter than paraglossae; inner margin with row of spine-like setae, increasing in length distally; apex with several short to long, robust setae; outer margin with row of spine-like setae; ventral surface with fine, simple scattered setae. Paraglossae laterally slightly undulated, distally truncated, and slightly bent inwards; apex with three rows of long, robust, distally pectinate setae; ventrally usually with several short, simple setae in distomedial area and one short, simple seta in proxolateral area; dorsally with few long, spine-like setae near inner margin. Labial palp with three segments, segment II with small to medium protuberance.

**Thorax. *Hind protoptera*** present, well developed.

**Figure 2.**
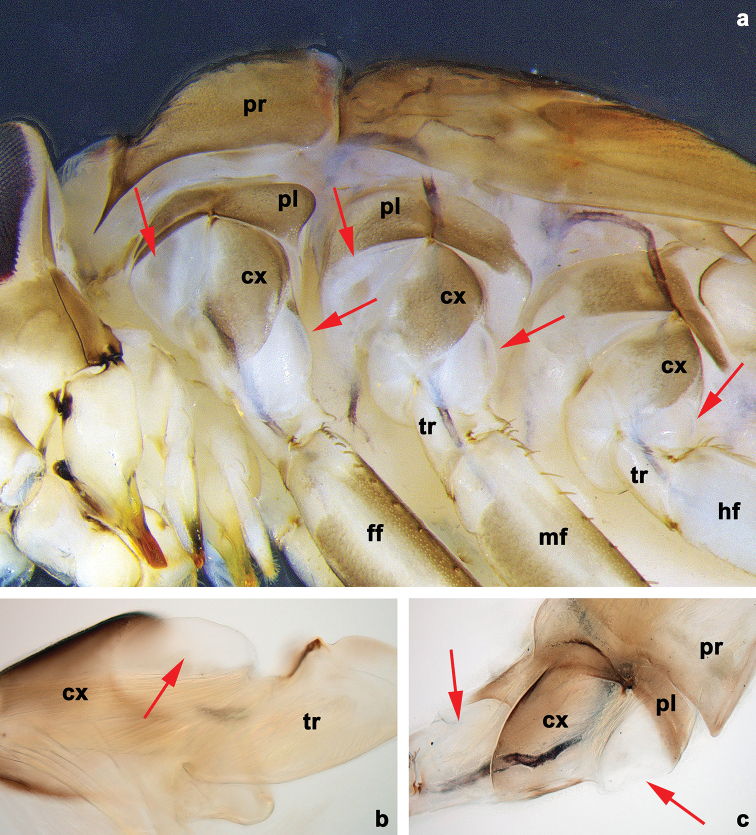
*Branchiobaetisjavanicus* comb. nov., larva **a** thorax, lateral view. *Branchiobaetisjoachimi* sp. nov., larva **b, c** foreleg. Abbreviations: cx, coxa; ff, forefemur; hf, hind femur; mf, middle femur; pl, pleurite; pr, pronotum; tr, trochanter.

**Figure 3.**
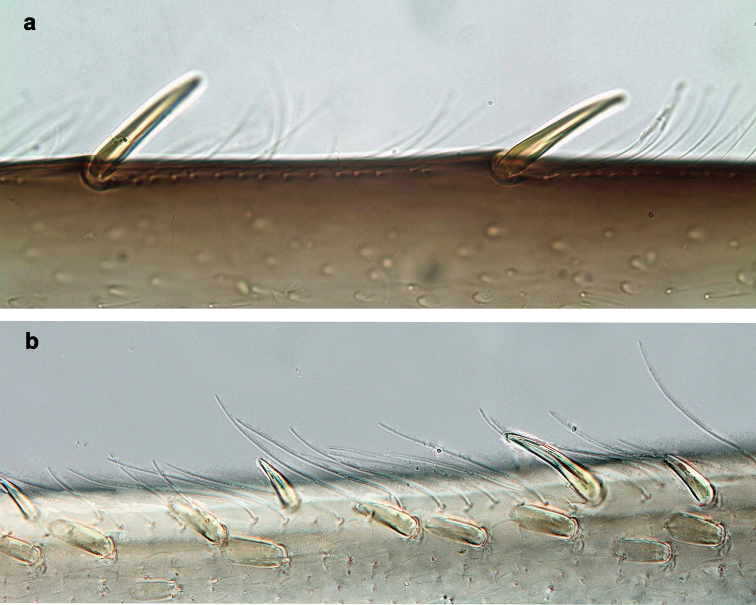
*Branchiobaetisjavanicus* comb. nov., larva **a** dorsal margin of foreleg. *Branchiobaetisjoachimi* sp. nov., larva **b** dorsal margin of foreleg.

***Foreleg*** (Figs 1b, 2a–c, 3a, b, 7a, d, g, k, 13a, 15a, d, e). ***Femur*** with row of medium to long, spine-like setae and additionally straight row of fine setae on dorsal margin; on apex short, stout setae on anterior and posterior side; femoral patch present, reduced and sometimes indistinct. Accessory gill on inner side of coxal articulation (between coxa and prosternum); bubble-like membranous swelling between coxa and trochanter and between coxa and pleurite (Figs 1b, 2a–c). ***Tibia*** with long patella-tibial suture in ¾ area; dorsal margin with row of short, stout setae and row of fine setae. ***Tarsus*** dorsally with row of short, stout setae, ventrally with row of curved, spine-like setae increasing in length distally. ***Claw*** robust, pointed, with one row of denticles; usually with one long, subapical seta (posterior seta sensu Kluge and Novikova 2014).

***Middle and hind leg*** (Figs 2a, 7b, c, e, f, h, i). As foreleg; femoral patch on middle leg also reduced, but well developed on hind leg; hind femur without apical setae on posterior side. Bubble-like membranous swelling on middle leg between coxa and trochanter and reduced between coxa and pleurite, on hind leg only between coxa and trochanter.

**Abdomen. *Tergalii*** (Figs 15g, 26a–e). Present on abdominal segments I–VII, dorsolaterally oriented; costal margin with minute denticles and short, fine setae; anal margin with minute denticles and alternating both short and long, fine setae.

***Paraproct*** (Fig. 15h). Posterior margin with stout spines; most species with short, stout, apically rounded setae near posterior margin. Cercotractor with numerous, small, marginal spines.

***Caudalii*** (Fig. 5e). Inner lateral margin of cerci and paracercus bilaterally with primary swimming setae.

**Figure 4.**
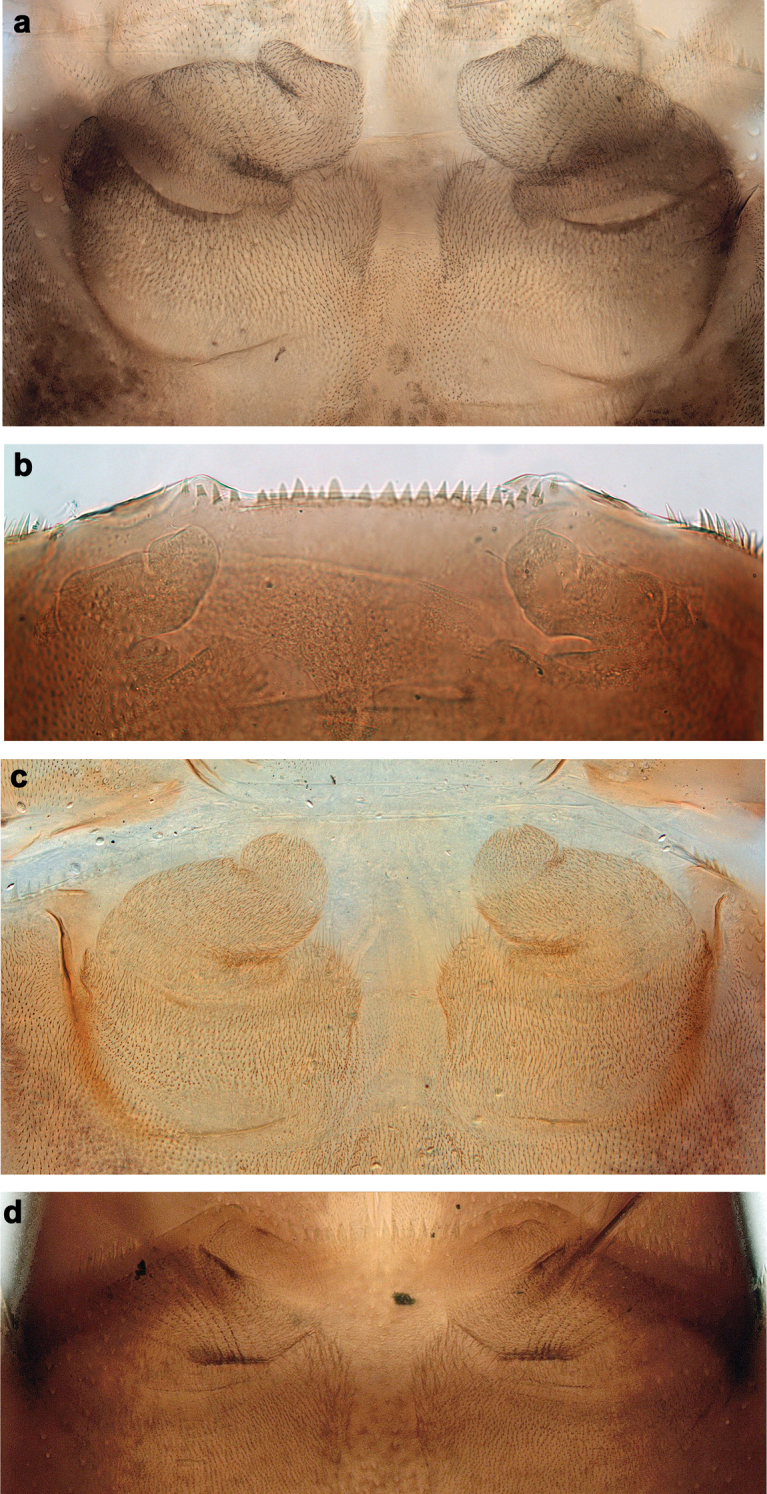
*Branchiobaetis* gen. nov., subimaginal gonostyli developing under cuticle of male last instar larva **a***B.javanicus* comb. nov. **b***B.sabahensis* comb. nov. (not yet fully developed) **c***B.aduncus* sp. nov. **d***B.joachimi* sp. nov.

***Larval protogonostyli*** (Fig. 10a) slightly projected; subimaginal gonostyli developing under cuticle of last instar larvae folded in the following way: segment II sharply bent towards middle, last segment sharply bent laterally (Figs 4a–d, 10a, b).

**Imago.** Forewing with double intercalary veins longer than distance between corresponding longitudinal vein; pterostigma with numerous cross veins (Fig. 9a, c). Hind wing with three longitudinal veins and well developed triangular costal projection (Fig. 9e). Imaginal gonostyli: segment I of gonostylus with projected blunt angle proximad of its middle; segment III short and triangular (Fig. 10d, e). Sterno-styligeral muscle clearly developed, but thin (Fig. 10f).

The imago is known from a single species (*B.javanicus* comb. nov.). Therefore, it is unclear, which of its characters are species-specific and which are generic (e.g., shape of turbinate eyes). Ulmer (1913, 1924) and Müller-Liebenau (1981) described imago and subimago and a complementary description is given below under *B.javanicus* comb. nov.

#### Distribution

**(Figs 27–29).** Indonesia (Sunda Islands, Kalimantan), Malaysia (Sabah), Philippines.

### ﻿1. Branchiobaetis javanicus

Taxon classificationAnimaliaEphemeropteraBaetidae

(Ulmer, 1913) comb. nov.

43A16986-0A59-5469-A762-297A15ADCE3B

Figs 1a, b , 3a , 5a–5 , 6 , 7 , 8 , 9 , 10 , 11d , 27b

Baetis javanicus : Ulmer 1913: 110 (♂ & ♀ imago); Müller-Liebenau 1981: 198 (♂ imago, larva); Sartori et al. 2016: 54 (syntypes locality).
Baetis javanica : Ulmer 1924: 52 (♂ & ♀ imago); Ulmer 1939: 523 (♂ imago, ♂ subimago, ♀ subimago); ibid.: 643 (larva).

#### Material examined.

**Type locality.** Indonesia • W. Java, Gedeh, Tjibodas; 1400 m; 24.-30.XII.1930; leg. M. A. Lieftinck; 2 ♀ larvae on slides; ZMH • Java, Cibodas; 6–11.VIII.2009; leg. N. Kluge & L. Sheyko; 4 ♂ subimagos with associated larval exuviae; [III](2)B2009, [III](7)B2009; 73 larvae; slides 7.XII.2021-1, 11.XII.2021-1, 24.XII.2021-1, 24.XII.2021-2, 24.XII.2021-3, 17.XII.2021-1; SPbU. **Other material.** Indonesia • Java, vic. Bogor, Mt. Sulak, Chiapus; 06°39'29"S, 106°44'55"E; 624 m; 24.II.2008; leg. S. Melnitsky; 1 ♂ imago; SPbU • Lombok, Mount Rinjani National Park; 25.IX.2009; leg. N. Kluge & L. Sheyko; 1 ♀ imago with associated larval and subimaginal exuviae; [XXXIX](1)2009; 34 larvae; SPbU • Java, Bogor, Ciliwung River, downstream of botanical garden; 06°35'32"S, 106°48'00"E; 235 m; 01.V.2010; leg. J.-M. Elouard; 1 larva on slide, GBIFCH00592476, 1 larva in alcohol, GBIFCH00592468; MZL • Java, Malang Batu Jalang, cascade, forest river; 07°54'52"S, 112°35'05"E; 570 m; 09.V.2010; leg. J.-M. Elouard; 2 larvae in alcohol, GBIFCH00592466, GBIFCH00592467; MZL.

#### B.cf.javanicus comb. nov. material examined.

Indonesia • Sumba, forest stream; 09°38'37"S, 119°40'56"E; 470 m; 27.IX.2011; leg. M. Balke; larva on slide; GBIFCH00592481; MZL; larva in alcohol; GBFCH00592463; MZL • Sumbawa, Batu Dulang, 10 mins to Tepal, forest stream; 08°35'52"S, 117°16'41"E; 860 m; 16.IX.2011; leg. M. Balke; 2 larvae on slides; GBIFCH00592479, GBIFCH00592480; MZL; 39 larvae in alcohol; GBIFCH00592462, GBIFCH00975593, GBIFCH00975594, GBIFCH00975604, GBIFCH00975605; MZL • Bali, Ubud, Sayan, Ayung River; 08°29'59"S, 115°14'35"E; 194 m; 20.IX.2011; leg. M. Balke; larva on slide; GBIFCH00592477; MZL • Bali, Ubud, Monkey River; 08°31'10"S, 115°15'18"E; 260 m; 16.V.2010; leg. J.-M. Elouard; larva on slide; GBIFCH00592478; MZL; 2 larvae in alcohol; GBIFCH00975611; MZL • Sumatra Barat, Universitas Andalas campus, forest stream; 00°54'40"S, 100°28'23"E; 360 m; 08.XI.2011; leg. M. Balke; 3 larvae on slides; GBIFCH00592474, GBIFCH00592475, GBIFCH00592502; MZL; 69 larvae in alcohol; GBIFCH00592489, GBIFCH00592501; GBIFCH00975582, GBIFCH00975583, GBIFCH00975595, GBIFCH00975596, GBIFCH00975597, GBIFCH00975603; MZL • Flores, Maumere region, river in garden land; 08°42'55"S, 124°04'24"E; 134 m; 21.IV.2012; leg. M. Balke; 2 larvae on slides; GBIFCH00592262, GBIFCH00592297; MZL; 18 larvae in alcohol; GBIFCH00592264, GBIFCH00592265, GBIFCH00975606; MZL.

#### Differential diagnosis.

**Larva.** Following combination of characters distinguish *B.javanicus* comb. nov. from other species of *Branchiobaetis* gen. nov.: A) labial palp segment II with triangular protuberance, segment III rather long (Müller-Liebenau 1981: fig. 1b); B) dorsal margin of fore femur with row of spine-like setae, basally dense and partly arranged in double row (Fig. 7a; Müller-Liebenau 1981: fig. 1k); C) posterior margin of tergite I smooth, without spines; posterior margins of tergites II–X with triangular spines, partly longer than wide (Fig. 7j), partly as long as wide; posterior margin of sternites: I–VI smooth, without spines; VII smooth or with few small spines; VIII with few spaced, small, blunt spines; IX with triangular spines; D) paraproct not expanded, with stout setae along posterior margin.

**Figure 5.**
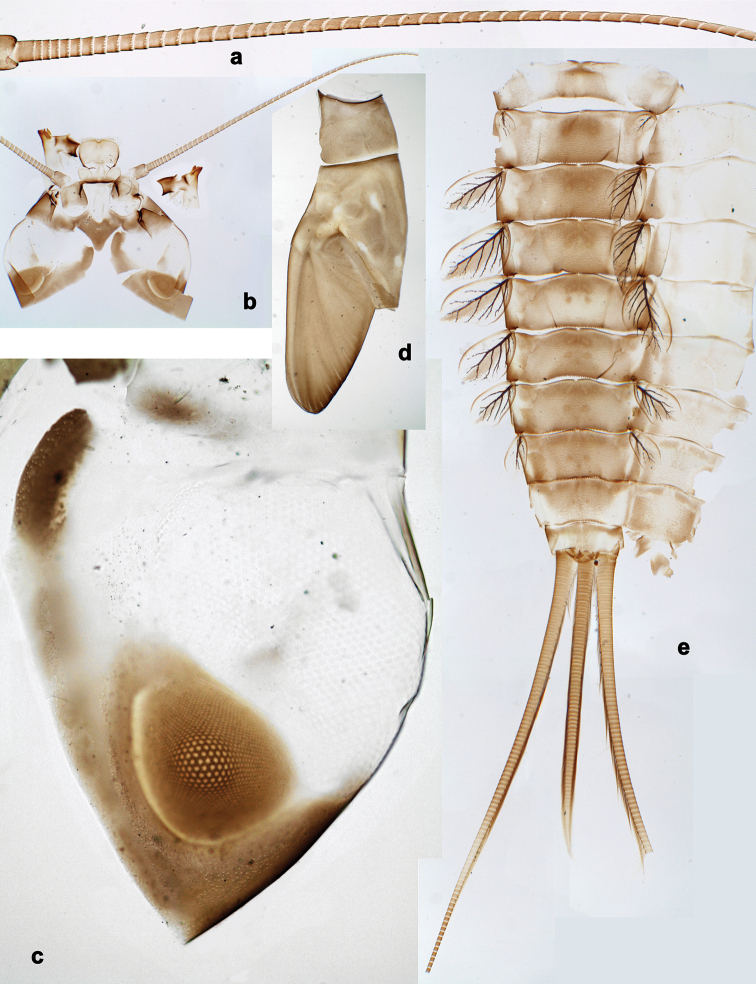
*Branchiobaetisjavanicus* comb. nov., exuviae of last instar male larva **a** portion of antenna **b** head **c** enlarged right eye and precursor of turbinate eye **d** left half of pronotum and mesonotum **e** abdomen.

#### Morphological features and their development.

**Figure 6.**
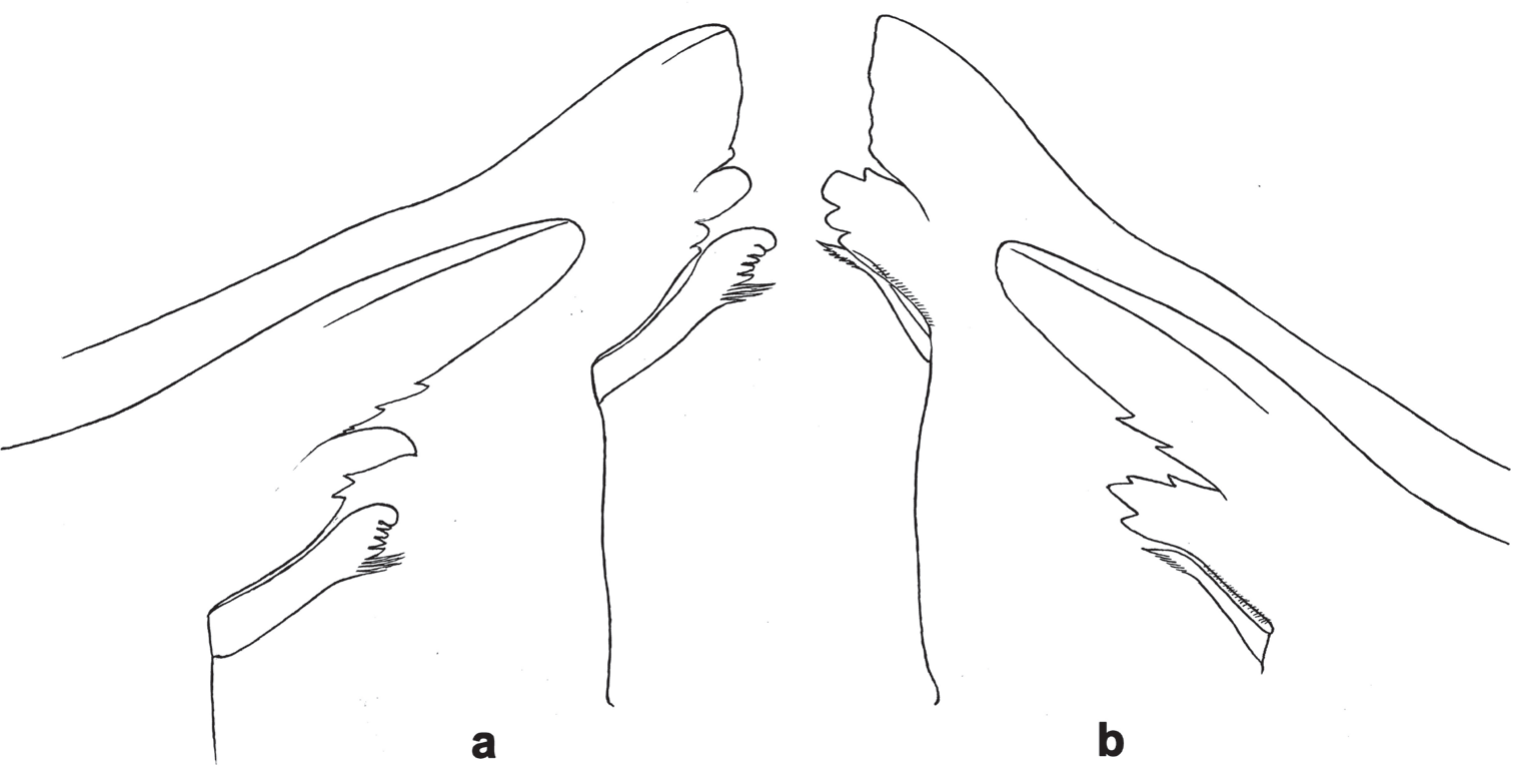
*Branchiobaetisjavanicus* comb. nov., larva **a, b** apices of left and right mandibles with mandibles of next instar developing inside them.

**Figure 7.**
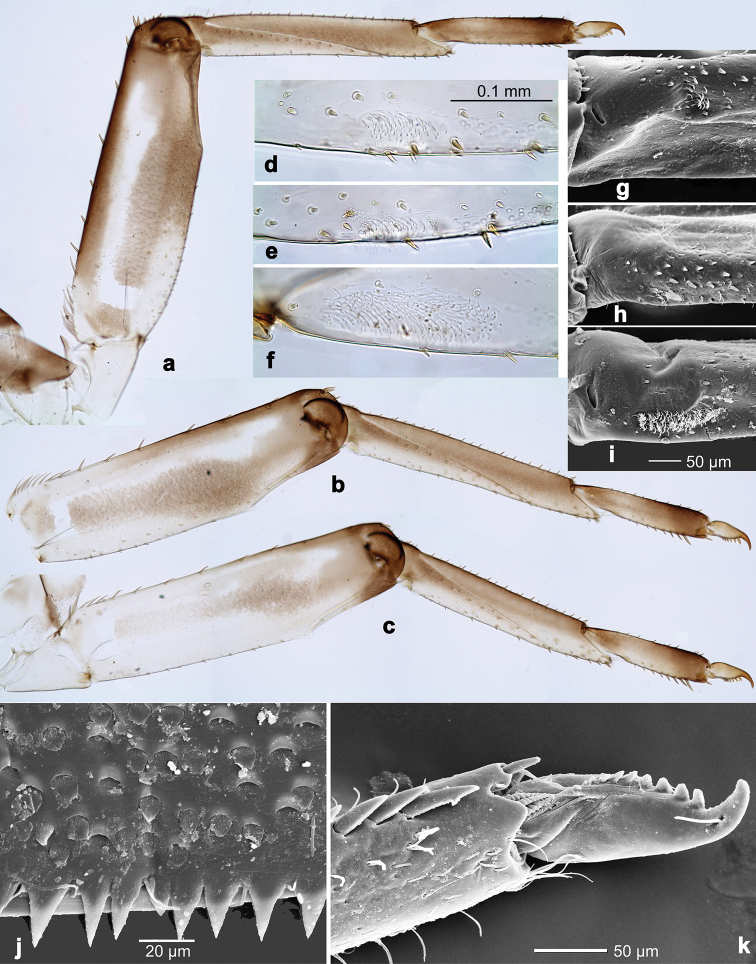
*Branchiobaetisjavanicus* comb. nov., larva **a–c** fore, middle, and hind legs **d–f** femoral patch of fore, middle and hind legs **g–i** femoral patch of fore, middle and hind legs **j** abdominal tergum **k** claw.

**Figure 8.**
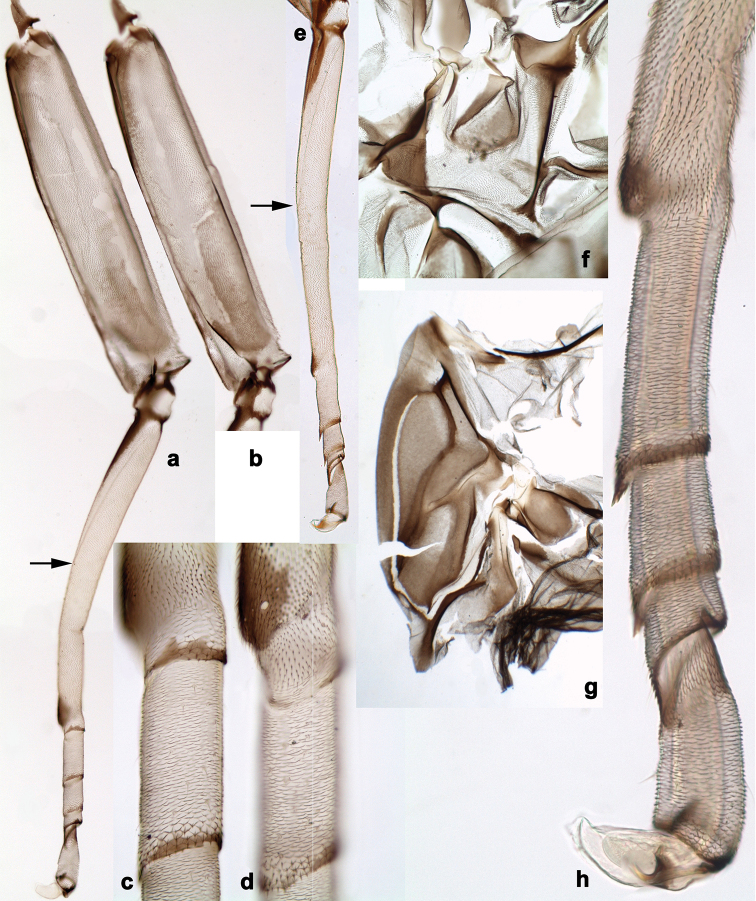
*Branchiobaetisjavanicus* comb. nov., subimagines **a–g** female subimaginal exuviae **a** foreleg, anterior view **b** fore femur, posterior view **c, d** base of fore tibia, anterior and posterior view **e** middle tibia **f** left mesopleuron with prealar and postsubalar sclerites **g** right part of mesonotum **h** middle tarsus of male subimago. Arrows show apex of patella-tibial suture.

**Figure 9.**
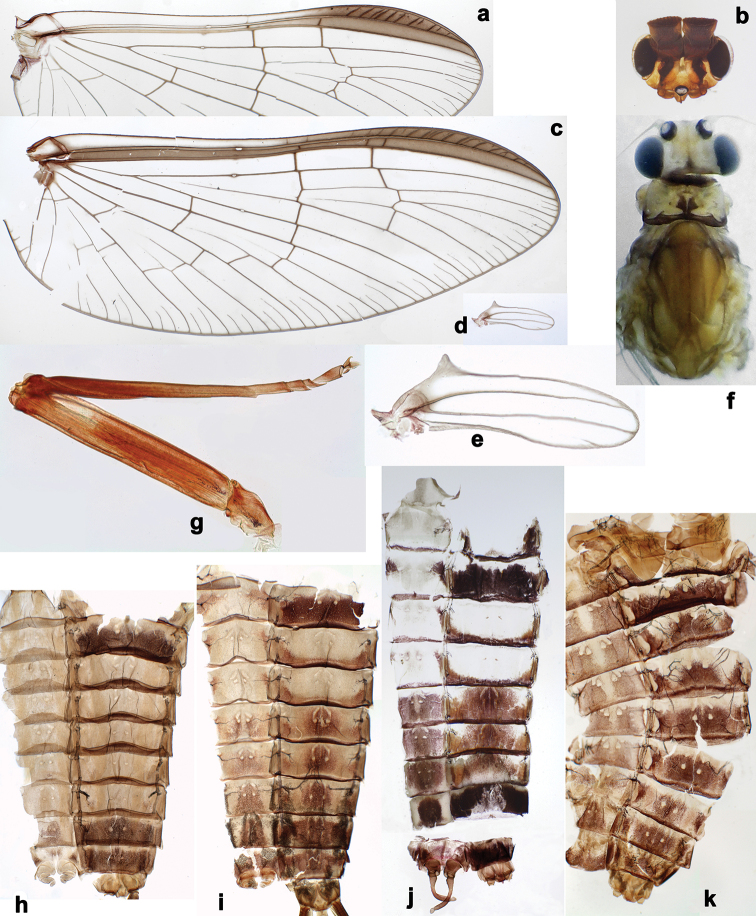
*Branchiobaetisjavanicus* comb. nov. **a** fore wing of male imago **b** head of male imago **c, d** fore and hind wing of female imago **e** hind wing enlarged **f** head and thorax of female imago **g** middle leg **h, i** male subimaginal abdomen extracted from larva **j** abdomen of male imago **k** abdomen of female imago.

**Figure 10.**
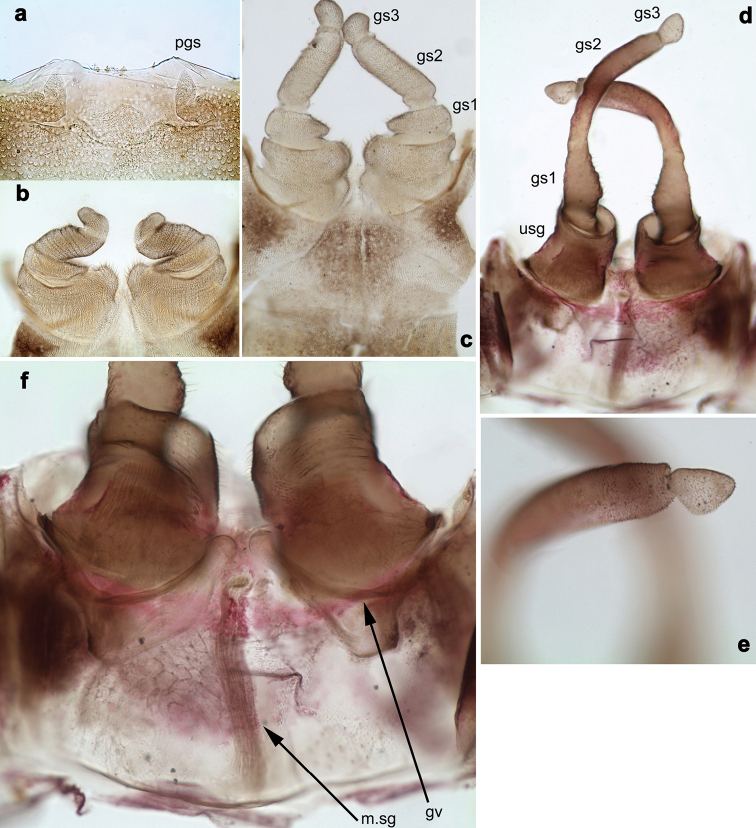
*Branchiobaetisjavanicus* comb. nov., male genitalia **a** subimaginal gonostyli crumpled under larval cuticle at earlier stage of development **b** subimaginal gonostyli extracted from larva starting to molt to subimago **c** genitalia of subimago, ventral view **d** genitalia of imago, ventral view **e** the same, apex of gonostylus **f** genitalia of imago, dorsal view. Abbreviations: gs1–gs3, segments of gonostyli; gv, gonovectis; m.gs, gonostylar muscle; m.sg, styligeral muscle; pgs, larval protogonostylus; usg, unistyliger.

Imagos and subimagos are described by Ulmer (1913, 1924). Müller-Liebenau correctly reported that hind wing has not two, but three veins (Fig. 9e; Müller-Liebenau 1981: fig. 2b). Larva is described by Ulmer (1939); larval characters are illustrated by Müller-Liebenau (1981: fig. 1). Here we give additional figures of larvae (Figs 1a, b, 3a, 5a–7k), subimagos (Fig. 8a–h), male imago (Figs 9a, b, j, 10d–f) and female imago (Fig. 9c–g, k).

***Turbinate eyes*.**Ulmer (1913, 1924) reported only colour of turbinate eyes (brown-grey), but not their shape. Turbinate eyes of male imago and subimago unusually small, cylindrical, with facetted surfaces round; facetted surface with approx. ten facets in diameter (Fig. 9b). In last instar male larva, precursors of the turbinate eyes representing a pair of reddish-brown maculae of egg-like shape; at middle of this macula, a smaller round area with well-expressed facets, approx. ten facets in diameter; peripheral area of the macula consists of very small and indistinct facets (Fig. 5c). Facetted surface of subimago and imago is developed from the round area, but not from the whole reddish brown macula.

***Larval mandibles*** (Fig. 6a, b). Incisors of left and right mandibles very long and parallel-sided (i.e., blade-like), with rounded apex and two small pointed denticles in proximal half.

N.B. Such shape of mandibular incisors is only visible when they are developed inside mandibles of the previous instar (Fig. 6a, b) and possibly just after the moult, before the mandibles are hardened and the larva starts to eat. After feeding, the incisors are worn and sometimes broken, so look much shorter (see outer lines of the same figures). Such worn mandibles are figured by Müller-Liebenau (1981: fig. 1e).

***Maxillary and sternal gills*** (Fig. 1a, b). Presence of small ventral tracheal gills not formerly reported. Presence of a pair of maxillary gills and a pair of fore coxal gills.

Each maxillary gill located on outer side of articulation between stipes and cardo; trachea penetrating into this gill, arising from paired tracheal stem which is more distally divided into branch penetrating into maxilla and branch penetrating into corresponding half of labium (Fig. 1a).

Each fore coxal gill located on inner side of coxal articulation, i.e., on the membrane between coxa and prosternum; trachea penetrating into this gill, arising from trachea going into foreleg; close to its base, trachea is divided into branch passing inside prosternum and branch penetrating into gill. Inside fore coxal gill, trachea widened, thin-walled and colourless (Fig. 1b).

***Patella-tibial suture*.** Patella-tibial suture present on all legs of larva, female subimago and female imago, including their fore legs (that is characteristic for Anteropatellata); greatly stretched along tibia: in larva reaching inner side of tibia in distal ¼ (Fig. 7a, c), in subimago and imago near middle of tibia (Figs 8a, e, 9g); in all stages patella-tibial suture barely reaching inner side of tibia, not crossing it.

***Femoral patch*.** Each larval leg with a femoral patch/field of minute curved setae on inner side of femur near its base (that is characteristic of Baetofemorata); femoral patch on hind leg large (Fig. 7f, i), but on fore and middle legs either much smaller (Fig. 7d, e), or indistinct (Fig. 7g, h).

**Figure 11.**
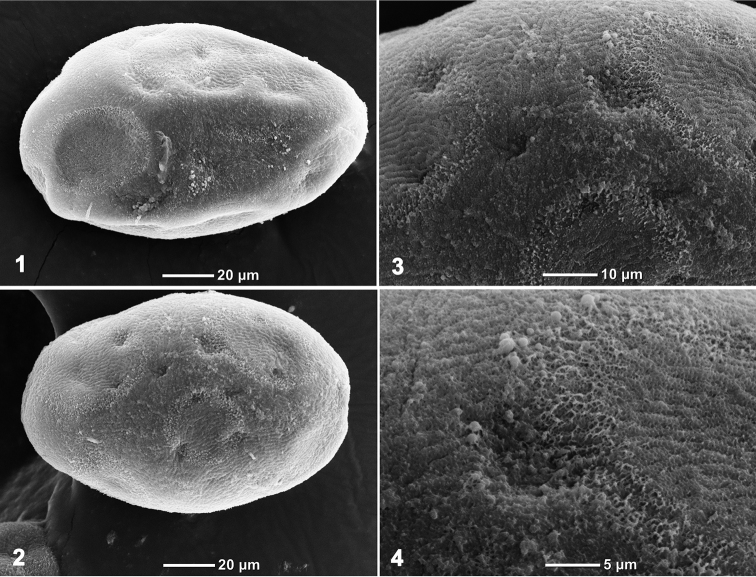
*Branchiobaetisjavanicus* comb. nov., eggs.

***Texture of subimaginal tarsi*** (Fig. 8c, d, h). In subimagos of both sexes, all tarsomeres covered with blunt microlepides; only very basal part of first tarsomere covered with microtrichia (like tibia), and apical parts of tarsomeres with pointed microlepides.

***Colouration of subimaginal cuticle*.** Head colourless, antennae brown. Pronotum brown. Mesonotum mostly brown (Fig. 8g). Thoracic pleura with brown and colourless areas (Fig. 8f). Legs mostly light brownish with dark brown markings on femur, tibia, and tarsus (Fig. 8a–e). Abdominal terga nearly uniformly brown, slightly darker laterally; sterna lighter; cerci lighter brownish.

***Colouration of abdomen of winged males*.** Abdominal colouration of male imago is adequately described by Ulmer (1913, 1924). It consists of contrasting colourless-white areas, vine-red areas and black areas, with sharply different colour patterns of the terga I–II, III–IV, V–VII, VIII–IX, and X, and sharply different colour patterns of the sterna I–IV, V–VII, VIII, and IX (Fig. 9j).

Abdominal colouration of subimago was briefly characterized by Ulmer (1924) as ‘Ähnlich der Imago, Segment III bis VII bräunlichgelb durchscheinend’. Among examined male subimagos reared from larvae or extracted from mature larvae, some individuals agree with this characteristic, i.e., their terga I–II and VIII–IX are dark brown, but terga III–VII and all sterna are uniformly light brownish (Fig. 9h); some individuals have terga and sterna III–VII differentiated somewhat approximating to that of imago (Fig. 9i).

***Gonostyli of male*.** Imaginal gonostyli with characteristic species-specific shape (Fig. 10d, e; Ulmer 1924: fig. 25): unistyliger (wrongly called ‘Glied I’ in Ulmer 1924) cylindrical, somewhat narrowed at middle; segment I of gonostylus (wrongly called ‘Glied II’ in Ulmer 1924) with projected blunt angle proximad of its middle; segment III of gonostylus (wrongly called ‘Glied IV’ in Ulmer 1924) short and triangular, i.e., apically widened and truncate.

N.B. When developing subimaginal gonostyli are bent under the larval cuticle, segment II of gonostylus is bent medially (as in other Baetofemorata), and segment III is sharply bent laterally, that is a peculiar feature of *Branchiobaetis* gen. nov. (Fig. 10a, b). In subimago freed from the larval cuticle, gonostyli retain features of their previous pose under larval cuticle, with segments II sharply bent medially and segments III somewhat bent laterally (Fig. 10c); the species-specific shape of segment III is present in imaginal stage only (Fig. 10d, e). A paradoxical feature is that segment III starts to develop as unusually long (Fig. 10a), later it is bent and pressed to the 2^nd^ segment (Fig. 10b), while subsequently it becomes shorter (Fig. 10c, d).

***Internal parts of male genitalia*.** Sterno-styligeral muscle developed, but slender; gonovectes S-shaped, i.e., arched, with apices curved cranially (Fig. 10f).

***Egg*** (Fig. 11a–d). Eggs irregularly oval, with irregularly situated shallow cavities, and surface of chorion rugose.

***Dimension*.** Size rather variable: fore wing length of male and female (and the general body length) varies from 6 mm to 10 mm; females usually larger than males.

#### Larval habitat.

Tergalii unable for rhythmical respiratory movements (as in other Baetungulata), and larvae are unable to live for a long time in stagnant water. Larvae are most abundant in fast streams with cold water.

#### Distribution

**(Fig. 27b).** Indonesia: Java, Lombok; B.cf.javanicus comb. nov. Indonesia: Sumatra, Bali, Sumba, Sumbawa, Flores.

### ﻿2. Branchiobaetis sabahensis

Taxon classificationAnimaliaEphemeropteraBaetidae

(Müller-Liebenau, 1984) comb. nov.

02F6F7AF-51A3-54BC-A2AF-C79154E564E4

Figs 12 , 13

Baetis sabahensis : Müller-Liebenau 1984b: 89; figs 3, 9, 14, 14a.

#### Material examined.

Malaysia • Sabah, Ranau; 14.–16.VII.1972; leg. G. F. Edmunds; ♂ larva on slide; SPbU • Sabah, Kundasang; 04.IX.1994; leg. S. C. Kang; ♂ larva on slide; SPbU.

#### B.cf.sabahensis comb. nov. material examined.

Indonesia • East Kalimantan, Bas. Malinau, River Seturan, loc. Seturan (2000-block 44–45), trib. Wok (Sungai Guang); 2°59'12"N, 116°33'11"E; 16.VI.2000; leg. P. Derleth & J.-L. Gattolliat; 3 larvae on slides; GBIFCH00592470, GBIFCH00592471, GBIFCH00592495; larva in alcohol; GBIFCH00270724; MZL • East Kalimantan, Bas. Malinau, River Seturan, loc. Seturan (2001-block 57), trib. Bengahau; 02°59'22"N, 116°30'46"E; 19.VIII.2000; leg. P. Derleth & R. Schlaepfer; larva on slide; GBIFCH00592494; larva in alcohol; GBIFCH00270710; MZL • East Kalimantan, Bas. Malinau, riv. Seturan, loc. Seturan (2001-block 57), trib. Benganau; 02°59'22"N, 116°30'46"E; 11.IV.2001; leg. P. Derleth & B. Feldmeyer; larva in alcohol; GBIFCH00270710; MZL.

**Figure 12.**
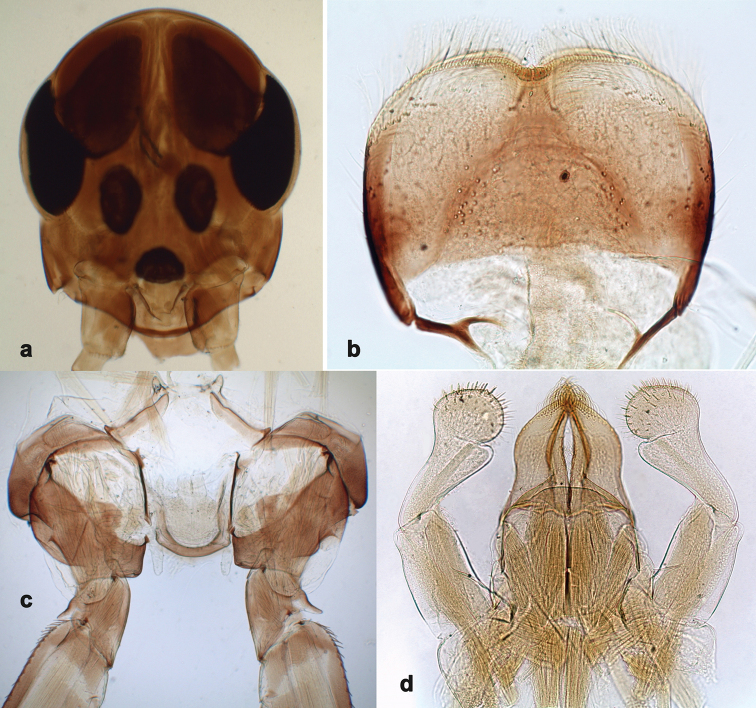
*Branchiobaetissabahensis* comb. nov., larva **a** head of male larva **b** labrum **c** prosternum and bases of forelegs, front view **d** labium.

#### Differential diagnosis.

**Figure 13.**
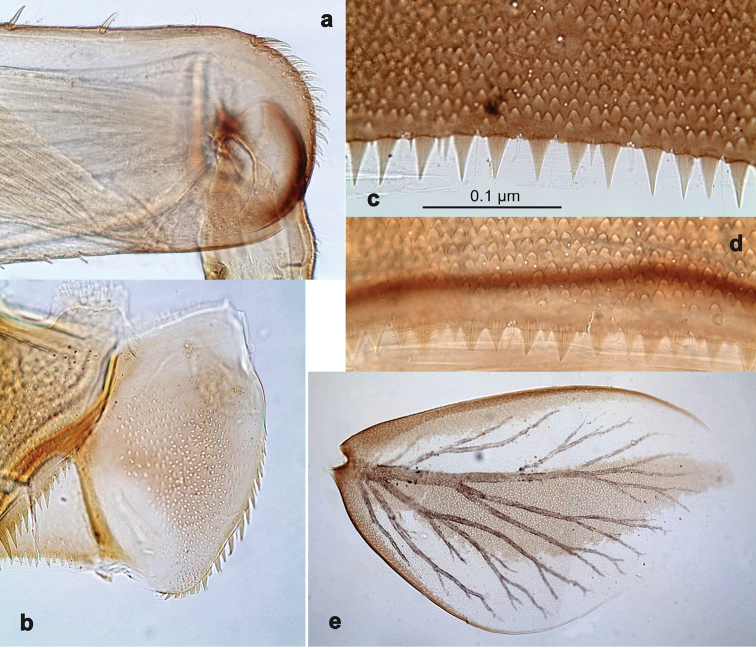
*Branchiobaetissabahensis* comb. nov., larva **a** apex of hind femur **b** paraproct **c** abdominal tergum IX **d** abdominal sternum VIII **e** tergalius.

**Larva.** Following combination of characters distinguish *B.sabahensis* comb. nov. from other species of *Branchiobaetis* gen. nov.: A) labial palp segment II with short, thumb-like protuberance; segment III rather short and wide, ca. 0.5× length of segment II, ca. 0.8× as long as width at base, ca. 0.7× as long as maximal width (Fig. 12d; Müller-Liebenau 1984b: fig. 3b); B) incisor of right mandible with ventral denticle (Müller-Liebenau 1984b: fig. 3e; C) dorsal margin of femur with row of ca. 15 long, spine-like setae; no additional row of short setae along margin; no short, stout setae on surface of femur (Fig. 13a; Müller-Liebenau 1984b: fig. 3k); D) posterior margin of tergite I smooth, without spines; posterior margins of tergites II–III with rounded or triangular spines, posterior margins of tergites IV–X with triangular spines (Fig. 13c; Müller-Liebenau 1984b: fig. 14); posterior margins of sternites: I–V smooth, without spines; VI smooth, without spines, or with some small, triangular spines; VII–IX with triangular or blunt spines (Fig. 13d).

**Imago.** Winged stages unknown. Judging from details revealed in last instar larva, turbinate eyes not narrowed; hind wing with costal projection; sterno-styligeral muscle present and thin.

#### Comments.

The original description of *Baetissabahensis* Müller-Liebenau, 1984 was based on larvae, and certain similarities of this species with *B.javanicus* were reported. The single argument to separate *B.sabahensis* from *B.javanicus*, was the statement that ”*Baetissabahensis* sp. nov. is the only species in the genus with coxal gills on the first pair of legs” (Müller-Liebenau 1984b: 92). Larva of *B.javanicus* was described and figured by the same author earlier (Müller-Liebenau 1981), but the coxal and maxillary gills had not been reported (see above).

Larva of *Branchiobaetissabahensis* comb. nov. can be separated from *B.javanicus* comb. nov. by the following characters: A) dense spines on abdominal sternite VIII (Fig. 13d); B) presence of only one or two stout setae on paraproct, or complete absence (Fig. 13b); C) incisor of right mandible with ventral denticle; D) labial palp segment III short and wide (Fig. 12d; see above).

Judging by precursors of turbinate eyes developed in last instar male larva, male imago of *B.sabahensis* comb. nov. should differ from *B.javanicus* comb. nov. by usual (not narrowed) turbinate eyes (Fig. 12a).

Branchiobaetiscf.sabahensis comb. nov. Specimens from Indonesia (Kalimantan) always have a series of stout setae along posterior margin of paraproct, contrary to specimens from Malaysia (Sabah). As there are no other differentiating characters to *B.sabahensis* comb. nov. from Malaysia (Sabah), we treat this population as B.cf.sabahensis comb. nov., waiting for genetic investigation of fresh material in the future.

#### Distribution

**(Fig. 27b).** Malaysia (Borneo: Sabah); B.cf.sabahensis comb. nov. Indonesia (Borneo: Kalimantan).

### ﻿3. Branchiobaetis aduncus sp. nov.

Taxon classificationAnimaliaEphemeropteraBaetidae

19C267A0-6F2B-5990-A401-21AEDD8A141A

https://zoobank.org/FE94DE11-B90B-42F7-81F8-FB99DA52F090

**Figure 14.**
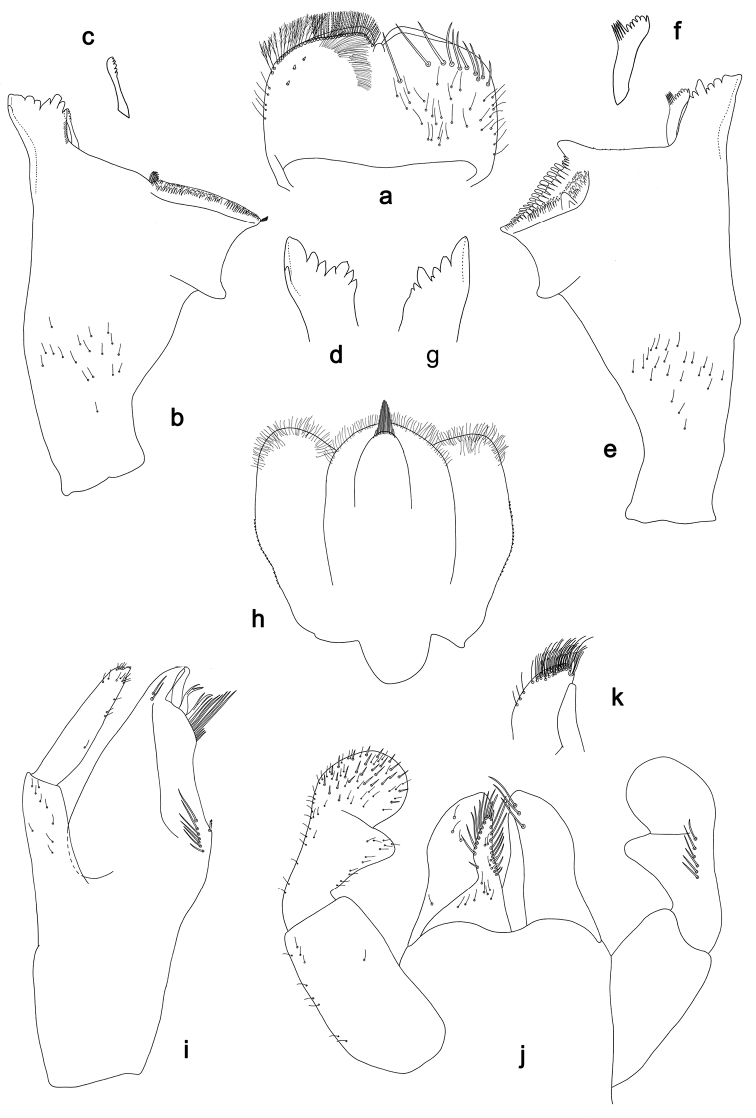
*Branchiobaetisaduncus* sp. nov., larva **a** labrum (left: ventral view, right: dorsal view) **b** right mandible **c** right prostheca **d** apex of right mandible **e** left mandible **f** left prostheca **g** apex of left mandible **h** hypopharynx and superlinguae **i** maxilla **j** labium (left: ventral view, right: dorsal view) **k** apex of paraglossa.

**Figure 15.**
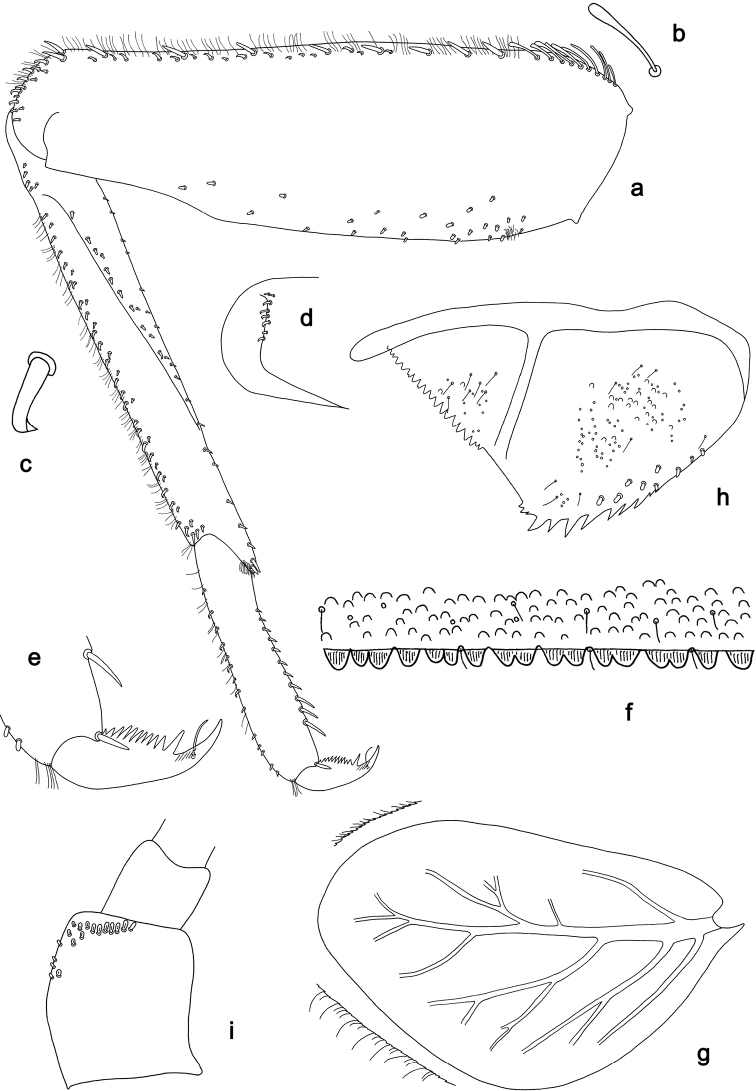
*Branchiobaetisaduncus* sp. nov., larva **a** foreleg **b** seta of femur basal dorsal margin **c** hook-like seta of leg dorsal margins **d** fore femur apex, posterior view **e** fore claw **f** tergum IV **g** tergalius IV **h** paraproct **i** base of antenna.

Figs 14 , 15 , 24a , 26a , 28a

#### Type material.

***Holotype*.** Indonesia • Sumatra, volcano Singgalang, River Caruak; 00°23'03"S, 100°21'24"E; 1640 m; 23.III.2014, leg. M. Gueuning; larva on slide; GBIFCH00422219; MZL. ***Paratypes*.** Same data as holotype; 1 larva on slide; GBIFCH00422126; MZL; 4 larvae in alcohol; GBIFCH00422185, GBIFCH00422194, GBIFCH00422203, GBIFCH00422209; MZL. Indonesia • Aceh, Mt. Leuser area, Kedah rainforest lodge; 03°58'36"S, 97°15'17"E; 1300 m, 3.–12.X.2013, leg. M. Balke; larva on slide; GBIFCH00515622; MZB (temporarily housed in MZL) • Sumatra Barat, Talang, 20 km south of Solok; 00°52'52"S, 100°37'23"E; 650 m; 25.V.2010; leg. J.-M. Elouard; larva on slide; GBIFCH00592486; MZL.

#### Differential diagnosis.

**Larva.** Following combination of characters distinguish *B.aduncus* sp. nov. from other species of *Branchiobaetis* gen. nov.: A) labial palp segment II with medium triangular protuberance, segment III apically rounded (Fig. 14j); B) incisor of right mandible with ventral denticle (Fig. 14b, d); C) dorsal margin of femur with row of medium, spine-like setae, basally longer and clavate; additional row of short, hook-like setae along margin (Fig. 15a–c); D) dorsal margin of tibia and tarsus with row of short, hook-like setae (Fig. 15a, c); E) posterior margin of tergites: I smooth, without spines; II–V rounded, wider than long; VI partly rounded, partly triangular; VII–IX triangular, narrower and longer towards last segment (Fig. 15f); posterior margins of sternites: I–VI smooth, without spines; VII–IX with small, spaced, triangular spines; F) paraproct with short, stout, apically rounded setae along posterior margin (Fig. 15h).

#### Description.

**Larva** (Figs 14, 15, 24a, 26a). Body length 7.0–8.1 mm. Caudalii broken. Antenna: ca. 2.5× as long as head length.

***Colouration*** (Fig. 24a). Head, thorax, and abdomen dorsally brown, ventrally light brown. Femur light brown, apically and dorsally along margin dark brown, with large, distomedial, dark brown spot; tibia light brown, basally along patella-tibial suture darker, tarsus dark brown. Caudalii light brown, primary swimming setae dark brown.

***Antenna*** (Fig. 15i). Scape distally and outside distolaterally with short, stout, apically rounded setae.

***Labrum*** (Fig. 14a). Length 0.6× maximum width. Submarginal arc of setae composed of nine or ten long, simple setae.

***Right mandible*** (Fig. 14b–d). Incisor blade-like with three denticles and a ventral denticle; kinetodontium with four denticles. Margin between prostheca and mola straight.

***Left mandible*** (Fig. 14e–g). Incisor blade-like with four denticles; kinetodontium with three denticles. Margin between prostheca and mola straight, with minute denticles towards subtriangular process.

Both mandibles with lateral margins slightly convex.

***Hypopharynx and superlinguae*** (Fig. 14h). Lingua as long as superlinguae. Lingua longer than broad; medial tuft of stout setae well developed. Superlinguae distally rounded; lateral margins rounded; fine, long, simple setae along distal margin.

***Maxilla*** (Fig. 14i). Galea-lacinia ventrally with two simple, apical setae under canines. Medially with one pectinate, spine-like seta and six or seven medium, simple setae. Maxillary palp approx. as long as galea-lacinia; palp segment II approx. as long as segment I; setae on maxillary palp fine, simple, scattered over surface of segments I and II.

***Labium*** (Fig. 14j, k). Inner margin of glossa with ca. nine spine-like setae, increasing in length distally; apex with one long, one medium and one short, robust setae; outer margin with ca. nine spine-like setae; Paraglossa with three short, simple setae in anteromedial area and one in posterolateral area; dorsally with three long, spine-like setae near inner margin. Labial palp with segment I 1.1× length of segments II and III combined. Segment I ventrally with short, fine, simple setae. Segment II with medium, triangular, distomedial protuberance; distomedial protuberance 0.5× width of base of segment III; ventral surface with short, fine, simple setae; dorsally with five or six spine-like setae near outer margin. Segment III apically rounded; length 0.8× maximum width; ventrally covered with short, spine-like, simple setae and short, fine, simple setae.

***Foreleg*** (Fig. 15a–e). Ratio of foreleg segments 1.3:1.0:0.5:0.2. ***Femur***. Length ca. 3× maximum width. Dorsal margin with row of 6–9 medium, curved, spine-like setae and basally 10–12 longer, clavate setae. Additional row of short, stout, hook-like setae along dorsal margin. Apex rounded, with pair of spine-like setae; short, stout, hook-like setae on anterior and posterior side. Short, stout, apically rounded setae scattered along ventral margin. ***Tibia***. Dorsal margin with two irregular rows of short, stout, hook-like setae. Surface with short, stout, hook-like setae along patella-tibial suture. Ventral margin with row of short, curved, spine-like setae, on apex a tuft of fine, simple setae. ***Tarsus***. Dorsal margin with row of short, stout, hook-like setae and row of fine, simple setae. ***Claw*** with one row of ten or eleven denticles, distal denticle much longer than other denticles.

***Terga*** (Fig. 15f). Surface with irregular rows of U-shaped scale bases and scattered fine, simple setae. Posterior margin of tergites: I smooth, without spines; II–V rounded, wider than long; VI partly rounded, partly triangular; VII–IX triangular, narrower and longer towards last segment. Posterior margins of sternites: I–VI smooth, without spines; VII–IX with small, spaced, triangular spines.

***Tergalii*** (Figs 15g, 26a). Tracheae extending from main trunk to inner and outer margins; with light brown band along main trunk of tracheae on anal side. Tergalius I 2/3 as long as segment II, tergalius IV as long as length of segments V and 1/3 VI combined, tergalius VII as long as length of segment VIII.

***Paraproct*** (Fig. 15h). Posterior margin with 12–16 stout spines. Short, stout, apically rounded setae near posterior margin. Surface scattered with scale bases, micropores and fine, simple setae.

#### Etymology.

Based on the Latin word *aduncus*, meaning hooked, with reference to the hook-like setae on the legs.

#### Distribution.

Indonesia: Sumatra (Fig. 28a).

#### Biological aspects.

The species was found at altitudes from 650 m to 1640 m, most specimens were collected in a forest stream with the following parameters: slope below 5%, width 1–3 m, depth 15–30 cm, velocity 0.2 m/s, water temperature 17 °C, pH 7, stream bed dominated by boulder, stones, and gravel.

### ﻿4. Branchiobaetis hamatus sp. nov.

Taxon classificationAnimaliaEphemeropteraBaetidae

C1A8A32D-99C4-57AA-874F-90BFDC0DF612

https://zoobank.org/4C505602-E896-4CA6-99BE-EFF79C82DBDF

**Figure 16.**
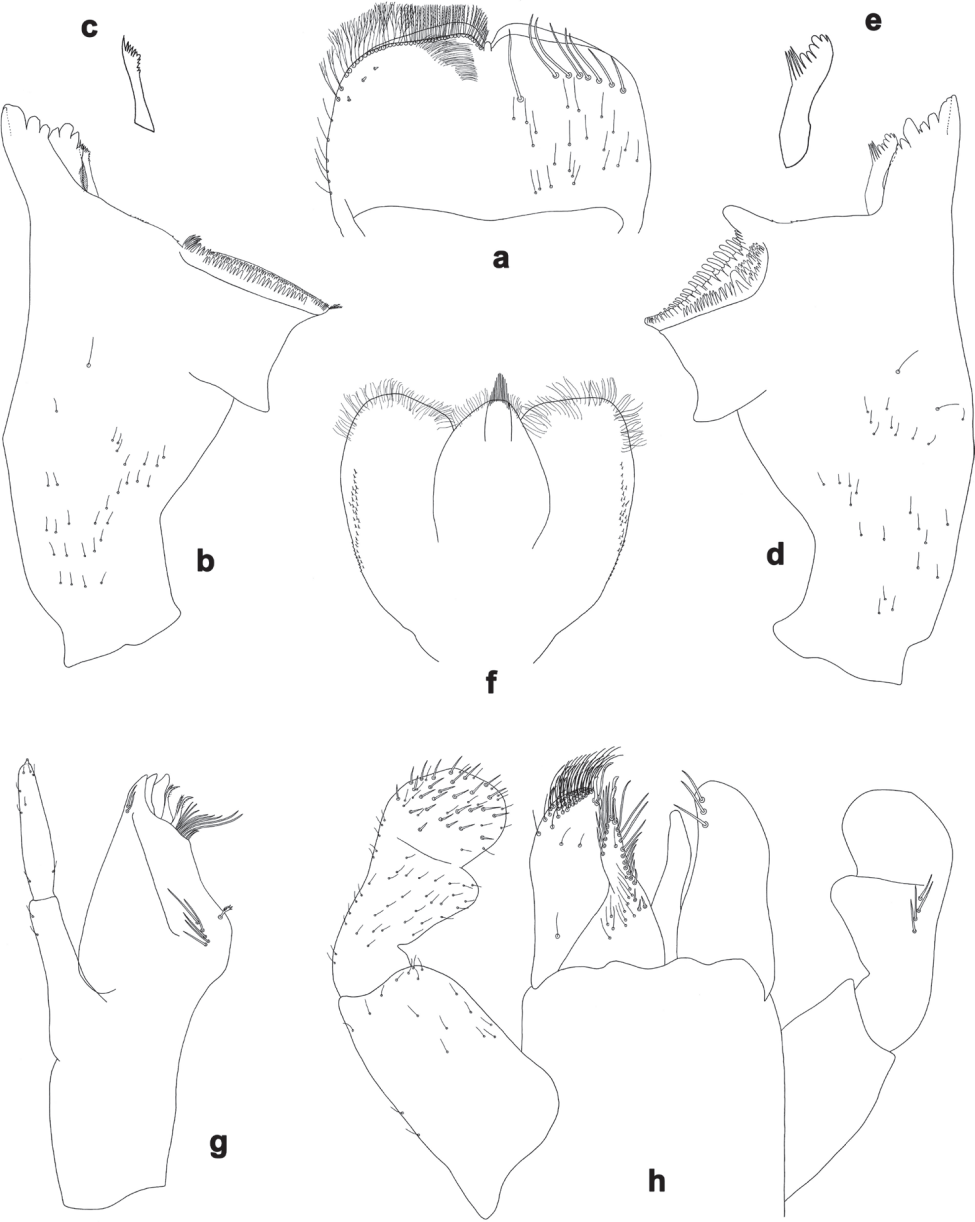
*Branchiobaetishamatus* sp. nov., larva **a** labrum (left: ventral view, right: dorsal view) **b** right mandible **c** right prostheca **d** left mandible **e** left prostheca **f** hypopharynx and superlinguae **g** maxilla **h** labium (left: ventral view, right: dorsal view).

**Figure 17.**
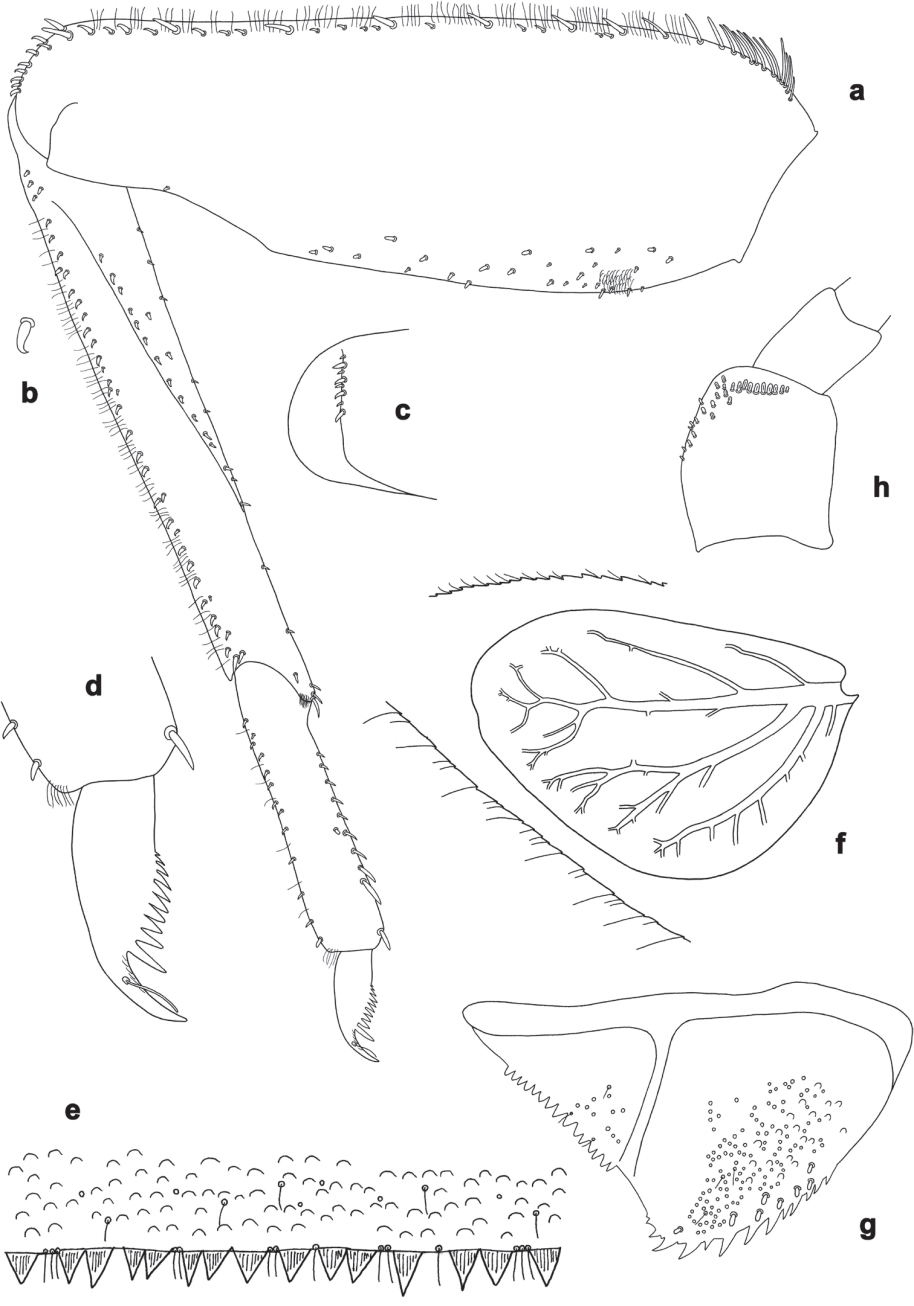
*Branchiobaetishamatus* sp. nov., larva **a** foreleg **b** hook-like seta of leg dorsal margins **c** fore femur apex, posterior view **d** fore claw **e** tergum IV **f** tergalius IV **g** paraproct **h** base of antenna.

Figs 16 , 17 , 24b , 26b , 28a

#### Type material.

***Holotype*.** Indonesia • Sumatra, volcano Talamau; River Pularian; 00°00'60"N, 100°00'01"E; 960 m; 01.IV.2014; leg. M. Gueuning: larva on slide; GBIFCH00422261; MZL. ***Paratypes*.** Same data as holotype; 2 larvae on slides; GBIFCH00422231, GBIFCH00422242; 20 larvae in alcohol; GBIFCH00422233, GBIFCH00422252, GBIFCH00422267, GBIFCH00422276, GBIFCH00422355, GBIFCH00422359, GBIFCH00422445, GBIFCH00422748, GBIFCH00422753, GBIFCH00422798, GBIFCH00422843, GBIFCH00423022, GBIFCH00975634, GBIFCH00975635; MZL. Indonesia • Sumatra, volcano Singgalang, River Sianok; 00°19'57"S, 100°19'19"E; 1150 m; 24.III.2014; leg. M. Gueuning; 2 larvae on slides; GBIFCH00422184, GBIFCH00423074; 13 larvae in alcohol; GBIFCH00422123, GBIFCH00422167, GBIFCH00422206, GBIFCH004208, GBIFCH00422215, GBIFCH00422216, GBIFCH00422224, GBIFCH00422797, GBIFCH00422889, GBIFCH00422938, GBIFCH01115975, GBIFCH01116020; MZL.

#### Differential diagnosis.

**Larva.** Following combination of characters distinguish *B.hamatus* sp. nov. from other species of *Branchiobaetis* gen. nov.: A) labial palp segment II with medium, rounded protuberance, segment III apically slightly pointed (Fig. 16h); B) incisor of right mandible with ventral denticle (Fig. 16b); C) dorsal margin of femur with row of medium, spine-like setae, basally longer and clavate; additional row of short, hook-like setae along margin (Fig. 17a, b); D) dorsal margin of tibia and tarsus with row of short, hook-like setae (Fig. 17a, b); E) posterior margin of tergites: I smooth, without spines; II–IX triangular, narrower and longer towards last segment (Fig. 17e); posterior margin of sternites: I–VII smooth, without spines; VIII–IX with small, spaced, triangular spines; F) tergalius IV apically slightly concave (Fig. 17f); G) paraproct with short, stout, apically rounded setae along posterior margin (Fig. 17g).

#### Description.

**Larva** (Figs 16, 17, 24b, 26b). Body length 6.8–8.5 mm. Cerci: broken. Paracercus: ca. 0.4× body length. Antenna: ca. 2.5× as long as head length.

***Colouration*** (Fig. 24b). Head, thorax and abdomen dorsally brown, abdominal segment X light brown; head, thorax and abdomen ventrally light brown, scape with dark brown spot at inner lateral side. Femur light brown, apically and dorsally along margin dark brown, with large, medial, dark brown spot; tibia light brown, tarsus dark brown in distal half. Caudalii light brown, cerci darker brown in area of ca. ½ of length, paracercus darker brown parallel to cerci; primary swimming setae dark brown.

***Precursors of turbinate eyes*** (Fig. 25c) in male last instar larvae representing a pair of subtriangular maculae; in the middle of this macula, a smaller, round, elevated area with well-expressed facets, approx. ten facets in diameter; peripheral area of the macula with indistinct facets.

***Antenna*** (Fig. 17h). Scape distally and outside distolaterally with short, stout, apically rounded setae.

***Labrum*** (Fig. 16a). Length 0.6× maximum width. Submarginal arc of setae composed of 7–10 long, simple setae.

***Right mandible*** (Fig. 16b, c). Incisor blade-like with three denticles and a ventral denticle; kinetodontium with four denticles. Margin between prostheca and mola straight, with minute denticles.

***Left mandible*** (Fig. 16d, e). Incisor blade-like with four denticles; kinetodontium with three denticles. Margin between prostheca and mola straight, with minute denticles towards subtriangular process.

Both mandibles with lateral margins slightly convex.

***Hypopharynx and superlinguae*** (Fig. 16f). Lingua as long as superlinguae. Lingua longer than broad; medial tuft of stout setae well developed. Superlinguae distally rounded; lateral margins rounded; fine, long, simple setae along distal margin.

***Maxilla*** (Fig. 16g). Galea-lacinia ventrally with two simple, apical setae under canines. Medially with one pectinate, spine-like seta and five or six medium, simple setae. Maxillary palp approx. as long as galea-lacinia; palp segment II approx. as long as segment I; setae on maxillary palp fine, simple, scattered over surface of segments I and II.

***Labium*** (Fig. 16h). Inner margin of glossa with 10–12 spine-like setae, increasing in length distally; apex with two long and one medium, robust setae; outer margin with six or seven spine-like setae; Paraglossa with two short, simple setae in anteromedial area and one in posterolateral area; dorsally with three long, spine-like setae near inner margin. Labial palp with segment I approx. as long as length of segments II and III combined. Segment I ventrally with short, fine, simple setae. Segment II with medium, rounded, distomedial protuberance; distomedial protuberance 0.3× width of base of segment III; ventral surface with short, fine, simple setae; dorsally with 4–8 spine-like setae near outer margin. Segment III apically slightly pointed; length 0.8× maximum width; ventrally covered with short, spine-like, simple setae and short, fine, simple setae.

***Foreleg*** (Fig. 17a–d). Ratio of foreleg segments 1.3:1.0:0.5:0.2. ***Femur***. Length ca. 3× maximum width. Dorsal margin with row of 7–9 medium, curved, spine-like setae and basally 10–15 longer, clavate setae. Additional row of short, stout, hook-like setae along dorsal margin. Apex rounded, with pair of spine-like setae; short, stout, hook-like setae on anterior and posterior side. Short, stout, apically rounded setae scattered along ventral margin. ***Tibia***. Dorsal margin with two irregular rows of short, stout, hook-like setae. On surface short, stout, hook-like setae along patella-tibial suture. Ventral margin with row of short, curved, spine-like setae, on apex a tuft of fine, simple setae. ***Tarsus***. Dorsal margin with row of short, stout, hook-like setae and row of fine, simple setae. ***Claw*** with one row of ten or eleven denticles, distal denticle much longer than other denticles.

***Terga*** (Fig. 17e). Surface with irregular rows of U-shaped scale bases and scattered fine, simple setae. Posterior margin of tergites: I smooth, without spines; II–IX triangular, narrower and longer towards last segment. Posterior margin of sternites: I–VII smooth, without spines; VIII–IX with small, spaced, triangular spines.

***Tergalii*** (Figs 17f, 26b). Tracheae extending from main trunk to inner and outer margins; with light brown band along main trunk of tracheae on anal side. Tergalius I 2/3 as long as segment II, tergalius IV as long as length of segments V and 1/2 VI combined, tergalius VII as long as length of segment VIII.

***Paraproct*** (Fig. 17g). Posterior margin with 11–16 stout spines. Short, stout, apically rounded setae near posterior margin. Surface scattered with scale bases, micropores and fine, simple setae.

#### Etymology.

Based on the Latin word *hamatus*, meaning hooked, with reference to the hook-like setae on the legs.

#### Distribution.

Indonesia: Sumatra (Fig. 28a).

#### Biological aspects.

The specimens were collected in two sites at altitudes of 940 m and 1150 m, with following physical conditions: slope 5–10%, width of stream 3–8 m, depth 1–50 cm, velocity 0.5 m/s–0.7 m/s, pH 8, stream bed dominated by boulder, stones and gravel or stones and sand respectively. One of the sites was strongly influenced by human activities, with lot of waste and brown water.

### ﻿5. Branchiobaetis joachimi sp. nov.

Taxon classificationAnimaliaEphemeropteraBaetidae

BCA880A2-7A79-5560-A2E8-7805778088EE

https://zoobank.org/442848A3-531A-428C-827B-99A2D71D2C78

**Figure 18.**
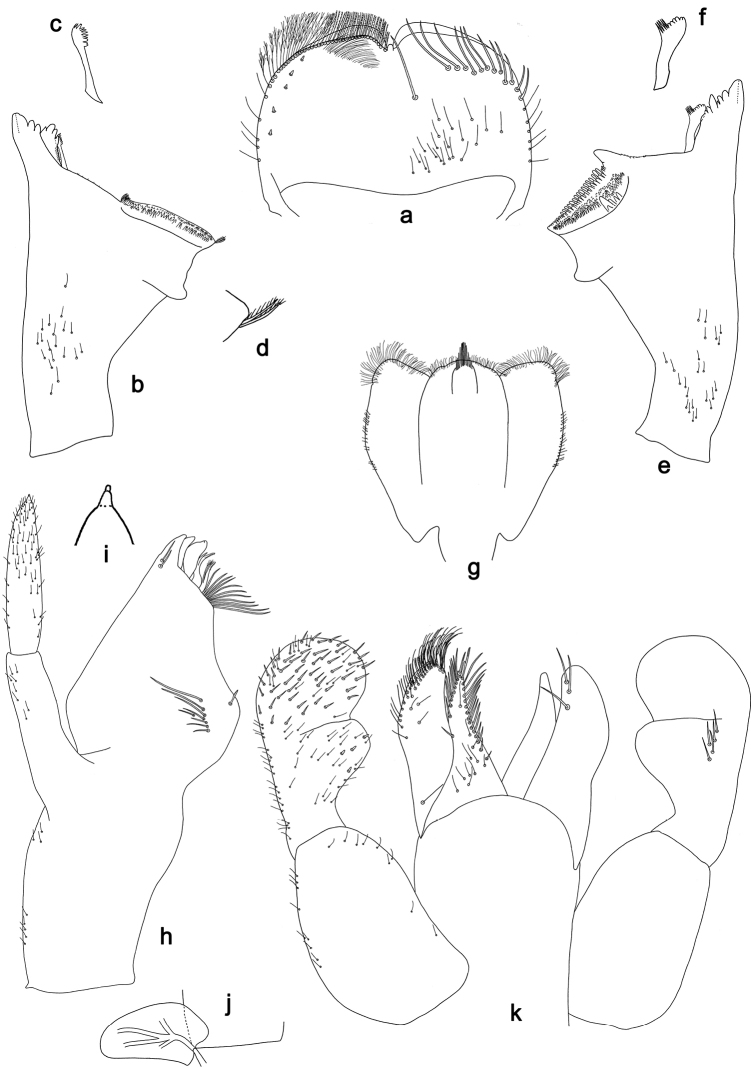
*Branchiobaetisjoachimi* sp. nov., larva **a** labrum (left: ventral view, right: dorsal view) **b** right mandible **c** right prostheca **d** mola apex of right mandible **e** left mandible **f** left prostheca **g** hypopharynx and superlinguae **h** maxilla **i** apex of maxillary palp **j** accessory gill between stipes and cardo of maxilla **k** labium (left: ventral view, right: dorsal view).

**Figure 19.**
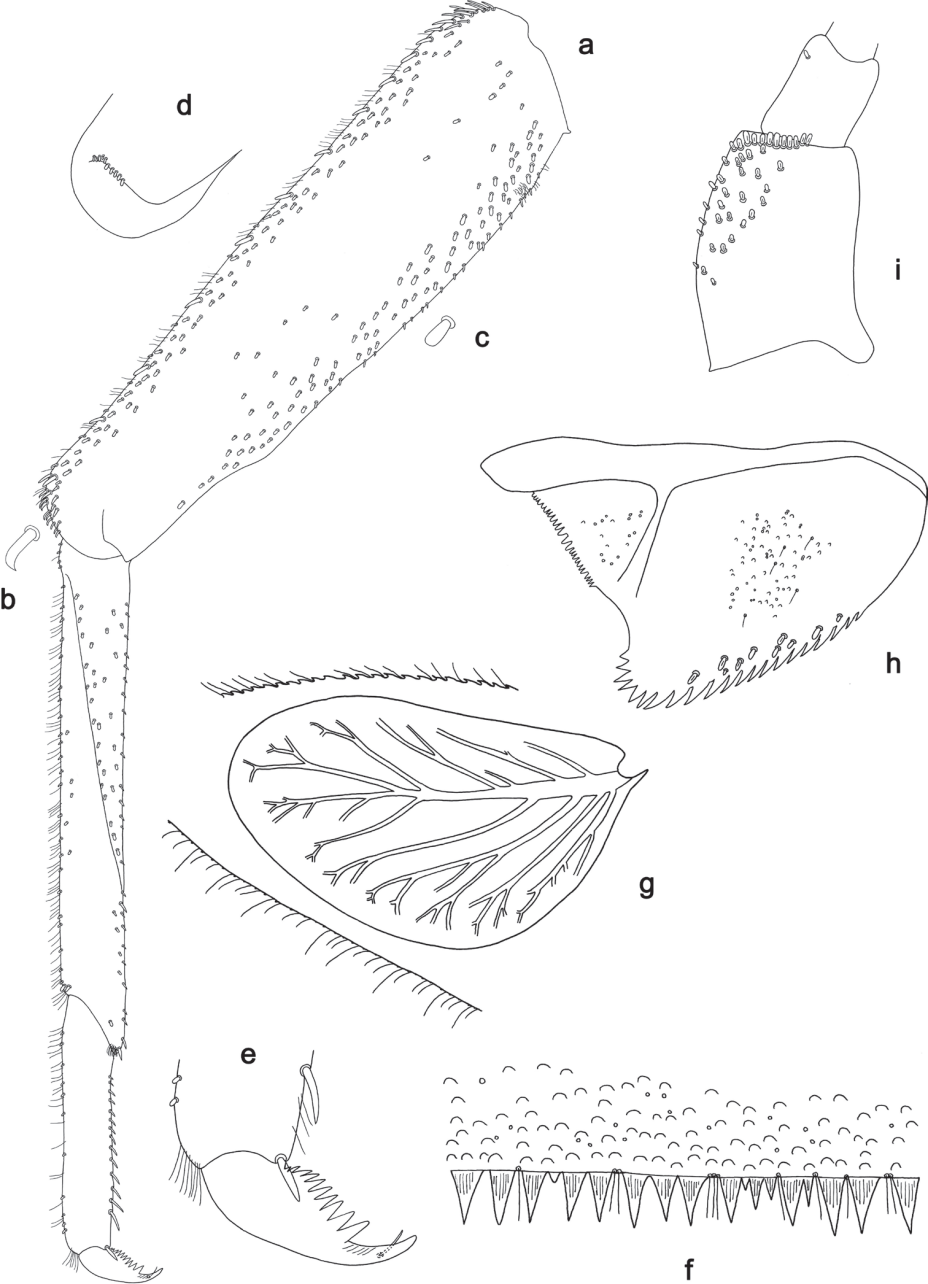
*Branchiobaetisjoachimi* sp. nov., larva **a** foreleg **b** hook-like seta of femur apex **c** seta on ventral surface of femur **d** fore femur apex, posterior view **e** fore claw **f** tergum IV **g** tergalius IV **h** paraproct **i** base of antenna.

Figs 18 , 19 , 24c, d , 26c , 28b

#### Type material.

***Holotype*.** Indonesia • Sumatra, volcano Marapi, East; 00°21'33"S, 100°30'42"E; 1205 m; 03.IV.2014; leg. M. Gueuning; larva on slide; GBIFCH00422405; MZL. ***Paratypes*.** Same data as holotype; 19 larvae in alcohol; GBIFCH00422228, GBIFCH00422235, GBIFCH00422238, GBIFCH00422241, GBIFCH00422254, GBIFCH00422266, GBIFCH00422402, GBIFCH00422440, GBIFCH00422489, GBIFCH00422709, GBIFCH00422844, GBIFCH00422887, GBIFCH00422932, GBIFCH00422977; MZL. Indonesia • Sumatra, volcano Sago, River Kobun; 00°22'33"S, 100°39'33"E; 1095 m; 19.III.2014; leg. M. Gueuning; 2 larvae on slide; GBIFCH00592506, GBIFCH00592507; 22 larvae in alcohol; GBIFCH00422152, GBIFCH00422166, GBIFCH00422173, GBIFCH00422222, GBIFCH00422226, GBIFCH00422227, GBIFCH00422253, GBIFCH00422256, GBIFCH00422258, GBIFCH00422266, GBIFCH00422268, GBIFCH00422270, GBIFCH00422663, GBIFCH00422708, GBIFCH00422754, GBIFCH00422928, GBIFCH00422979, GBIFCH00423113; MZL • Sumatra, volcano Sago, River Tampo; 00°22'20"S, 100°41'45"E; 960 m; 20.III.2014; leg. M. Gueuning; 8 larvae in alcohol; GBIFCH00422232, GBIFCH00422239, GBIFCH00422249, GBIFCH00422250, GBIFCH00422255, GBIFCH00422265, GBIFCH00422619, GBIFCH00423112; MZL • Sumatra, volcano Sago, River Tampo; 00°20'37"S, 100°41'02"E; 1255 m; 21.III.2014; leg. M. Gueuning; 12 larvae in alcohol; GBIFCH00422230, GBIFCH00422236, GBIFCH00422237, GBIFCH00422243, GBIFCH00422251, GBIFCH00422264, GBIFCH00422883, GBIFCH00423026, GBIFCH00423068, GBIFCH00423116; MZL • Sumatra, volcano Sago, River Kaligain; 00°18'01"S, 100°40'08"E; 1040 m; 05.IV.2014; leg. M. Gueuning; 1 larva on slide; GBIFCH00592525; 22 larvae in alcohol; GBIFCH00422229, GBIFCH00422234, GBIFCH00422244, GBIFCH00422246, GBIFCH00422259, GBIFCH00422263, GBIFCH00422304, GBIFCH00422441, GBIFCH00422442, GBIFCH00422443, GBIFCH00422659, GBIFCH00975612, GBIFCH00975613; MZL • Sumatra, volcano Singgalang, River Airjernih; 00°24'07"S, 100°16'44"E; 840 m; 25.III.2014; leg. M. Gueuning; 1 larva on slide; GBIFCH00422159; 7 larvae in alcohol; GBIFCH00422052, GBIFCH00422142, GBIFCH00422157, GBIFCH00422160, GBIFCH00422618, GBIFCH00422752, GBIFCH00423024; MZL • Sumatra, volcano Singgalang, River Magyih; 00°23'33"S, 100°16'34"E; 845 m; 25.III.2014; leg. M. Gueuning; 1 larva on slide; GBIFCH00422211; 5 larvae in alcohol; GBIFCH00422081, GBIFCH00422154, GBIFCH00422198, GBIFCH00422201, GBIFCH00422217; MZL • Sumatra, volcano Singgalang, River Magyih; 00°22'50"S, 100°17'39"E; 1075 m; 26.III.2014; leg. M. Gueuning; 3 larvae in alcohol; GBIFCH00422098, GBIFCH00422168, GBIFCH00422221; MZL • Sumatra, volcano Singgalang, River Sianok; 00°19'57"S, 100°19'19"E; 1150 m; 24.03.2014; leg. M. Gueuning; 1 larva in alcohol; GBIFCH00422248; MZL.

#### Other material.

Indonesia • Sumatra Barat, Bukit Barisan, above Padang, creek; 00°56'44"S, 100°32'44"E; 1047 m; 08.XI.2011; leg. M. Balke (UN3); 3 larvae on slides; GBIFCH00592472, GBIFCH00592473, GBIFCH00592505; 17 larvae in alcohol; GBIFCH00975598, GBIFCH00975599, GBIFCH00975602, GBIFCH00980897, GBIFCH00980898; MZL.

#### Differential diagnosis.

**Larva.** Following combination of characters distinguish *B.joachimi* sp. nov. from other species of *Branchiobaetis* gen. nov.: A) labial palp segment II with short, broad, rounded protuberance, with few small, stout, simple setae on protuberance; segment III apically rounded (Fig. 18k); B) dorsal margin of femur with row of medium, spine-like setae; many short, stout, apically rounded setae along dorsal margin; same type of setae scattered on surface and along ventral margin (Fig. 19a, c); C) posterior margin of tergites: I with triangular, pointed spines or short, triangular, blunt spines; II–IX with triangular, pointed spines, longer than wide (Fig. 19f); posterior margin of sternites: I–IV smooth, without spines; V with small, spaced, triangular spines; VI–IX with triangular spines; D) paraproct with short, stout, apically rounded setae along posterior margin (Fig. 19h).

#### Description.

**Larva** (Figs 18, 19, 24c, d, 26c). Body length 7.9–9.6 mm. Cerci: ca. 0.6× body length. Paracercus: ca. 0.6× cerci length. Antenna: ca. 2.5× as long as head length.

***Colouration*** (Fig. 24c, d). Head, thorax and abdomen dorsally brown or grey-brown, with pattern as in Fig. 24c or 24d; head, thorax and abdomen ventrally light brown or light grey, abdominal segment IX laterally with dark brown streaks. Femur light brown or light grey, apically and dorsally along margin dark brown, with large, distomedial, dark brown spot; tibia light brown or grey, basally dark brown along patella-tibial suture; tarsus light brown or grey, dark brown in distal half. Caudalii light grey-brown, darker brown area on ca. ½ of cerci length; primary swimming setae dark brown.

***Precursors of turbinate eyes*** in male last instar larvae representing a pair of brownish, egg-shaped maculae; in the middle of this macula, a smaller, round, elevated area with well-expressed facets, ca. 14 facets in diameter; peripheral area of the macula with indistinct facets (Figs 24c, d, 25d).

***Antenna*** (Fig. 19i). Scape distally and outside distolaterally with short, stout, apically rounded setae.

***Labrum*** (Fig. 18a). Length 0.6× maximum width. Submarginal arc of setae composed of 10–12 long, simple setae.

***Right mandible*** (Fig. 18b–d). Incisor blade-like with three denticles; kinetodontium with four denticles. Margin between prostheca and mola straight, with minute denticles.

***Left mandible*** (Fig. 18e, f). Incisor blade-like with four denticles; kinetodontium with three denticles. Margin between prostheca and mola straight, with minute denticles towards subtriangular process.

Both mandibles with lateral margins almost straight.

***Hypopharynx and superlinguae*** (Fig. 18g). Lingua as long as superlinguae. Lingua longer than broad; medial tuft of stout setae well developed. Superlinguae distally rounded; lateral margins rounded; fine, long, simple setae along distal margin.

***Maxilla*** (Fig. 18h, i). Galea-lacinia ventrally with two simple, apical setae under canines. Medially with one pectinate, spine-like seta and 8–11 short to long, simple setae, not all in a row. Maxillary palp slightly longer than galea-lacinia; palp segment II ca. 1.2× as long as segment I; setae on maxillary palp fine, simple, scattered over surface of segments I and II.

***Labium*** (Fig. 18k). Inner margin of glossa with ca. 14 spine-like setae, increasing in length distally; apex with two long and one medium, robust setae; outer margin with approx. nine spine-like setae; Paraglossa with three or four short, simple setae in anteromedial area and one in posterolateral area; dorsally with three long, spine-like setae near inner margin. Labial palp with segment I approx. as long as length of segments II and III combined. Segment I ventrally with short, fine, simple setae. Segment II with short, broad, rounded, distomedial protuberance; distomedial protuberance 0.2× width of base of segment III; ventral surface with short, fine, simple setae and some short, stout, simple setae; dorsally with 4–6 spine-like setae near outer margin. Segment III about semi-circular, apically rounded; length 0.8× maximum width; ventrally covered with short, spine-like, simple setae and short, fine, simple setae.

***Foreleg*** (Fig. 19a–e). Ratio of foreleg segments 1.5:1.0:0.5:0.2. ***Femur***. Slender, length ca. 4× maximum width. Dorsal margin with row of 8–10 medium, curved, spine-like setae and basally 8–10 setae of same type, but standing denser and in more than one row. Further row of short, stout, hook-like setae on distal half of margin. Additionally many short, stout, apically rounded setae along dorsal margin. Same type of setae also on surface and many scattered along ventral margin. Apex rounded, with pair of medium, curved, spine-like setae and many short, hook-like setae. ***Tibia***. Dorsal margin with row of short, stout, apically rounded setae. On surface same type of setae along patella-tibial suture. Ventral margin with row of short, curved, spine-like setae, on apex a tuft of fine, simple setae. ***Tarsus***. Dorsal margin with row of short, stout setae and row of fine, simple setae. ***Claw*** with one row of ten denticles.

***Terga*** (Fig. 19f). Surface with irregular rows of U-shaped scale bases and scattered micropores. Posterior margin of tergites: I with triangular, pointed spines or short, triangular, blunt spines; II–IX with triangular, pointed spines, longer than wide. Posterior margin of sternites: I–IV smooth, without spines; V with small, spaced, triangular spines; VI–IX with triangular spines.

***Tergalii*** (Figs 19g, 26c). Tracheae extending from main trunk to inner and outer margins; with light brown band along main trunk of tracheae on anal side. Tergalius I 3/4 as long as segment II, tergalius IV as long as length of segments V and 1/2 VI combined, tergalius VII as long as length of segments VIII and ¼ IX combined.

***Paraproct*** (Fig. 19h). Posterior margin with 18–21 stout spines. Short, stout, apically rounded setae near posterior margin. Surface scattered with scale bases, micropores and fine, simple setae.

#### Etymology.

Dedicated to Joachim Kaltenbach, the late father of the first author.

#### Distribution.

Indonesia: Sumatra (Fig. 28b).

#### Biological aspects.

The specimens were collected on altitudes between 845 m and 1270 m, in the following physical conditions: slope 5–10%, width of stream 0.2–8 m, depth 7–40 cm, velocity 0.3 m/s–0.8 m/s, pH 6.5–7.5, stream bed dominated by boulder, stones and gravel and only exceptionally by sand and silt. Some of the sites were influenced or polluted by human activities.

### ﻿6. Branchiobaetis minangkabau sp. nov.

Taxon classificationAnimaliaEphemeropteraBaetidae

9634FA9A-0BC5-5E26-8ACF-75E8647C7F93

https://zoobank.org/B434954C-6136-4B65-9810-804B6B5581C9

**Figure 20.**
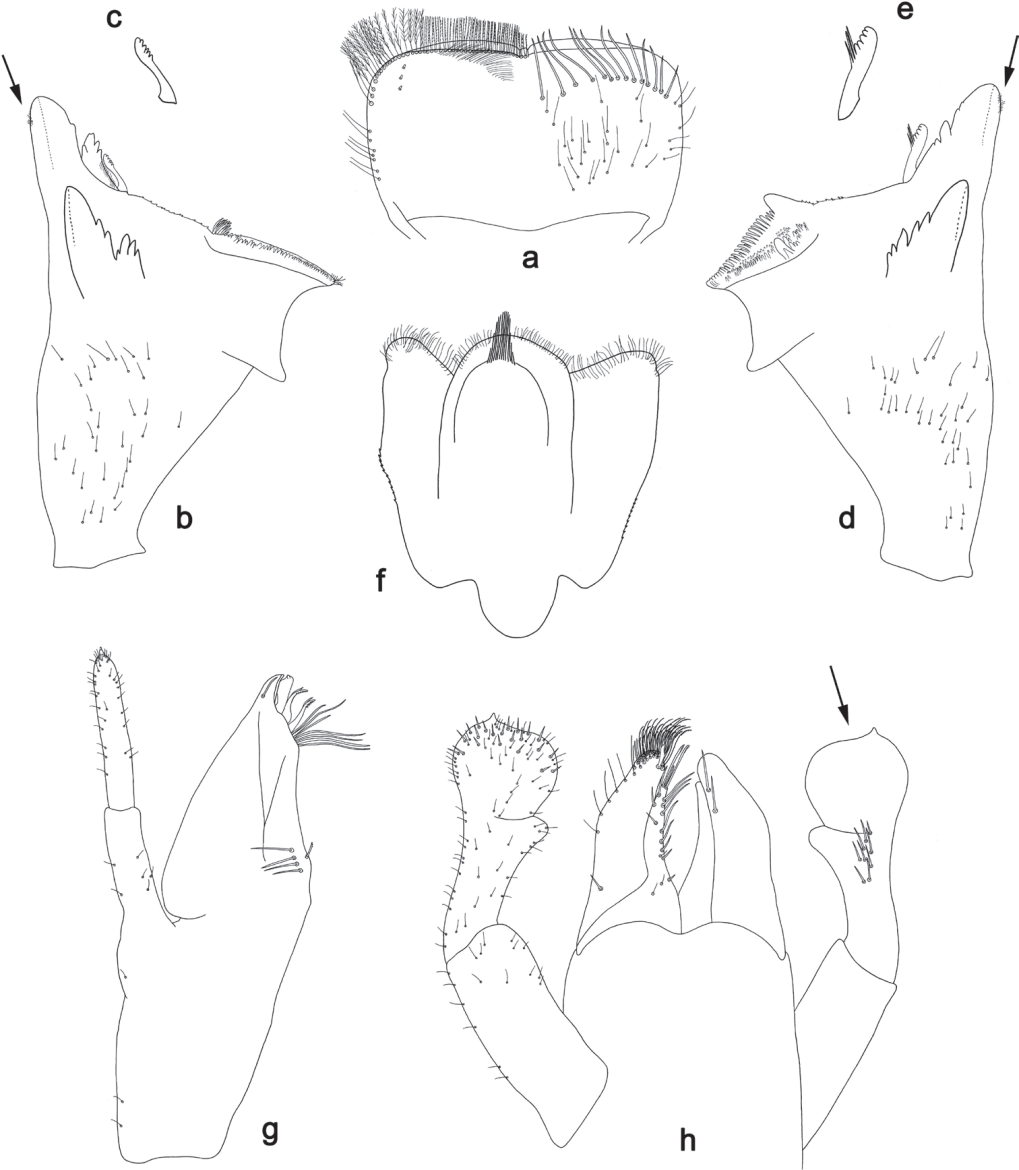
*Branchiobaetisminangkabau* sp. nov., larva **a** labrum (left: ventral view, right: dorsal view) **b** right mandible **c** right prostheca **d** left mandible **e** left prostheca **f** hypopharynx and superlinguae **g** maxilla **h** labium (left: ventral view, right: dorsal view).

**Figure 21.**
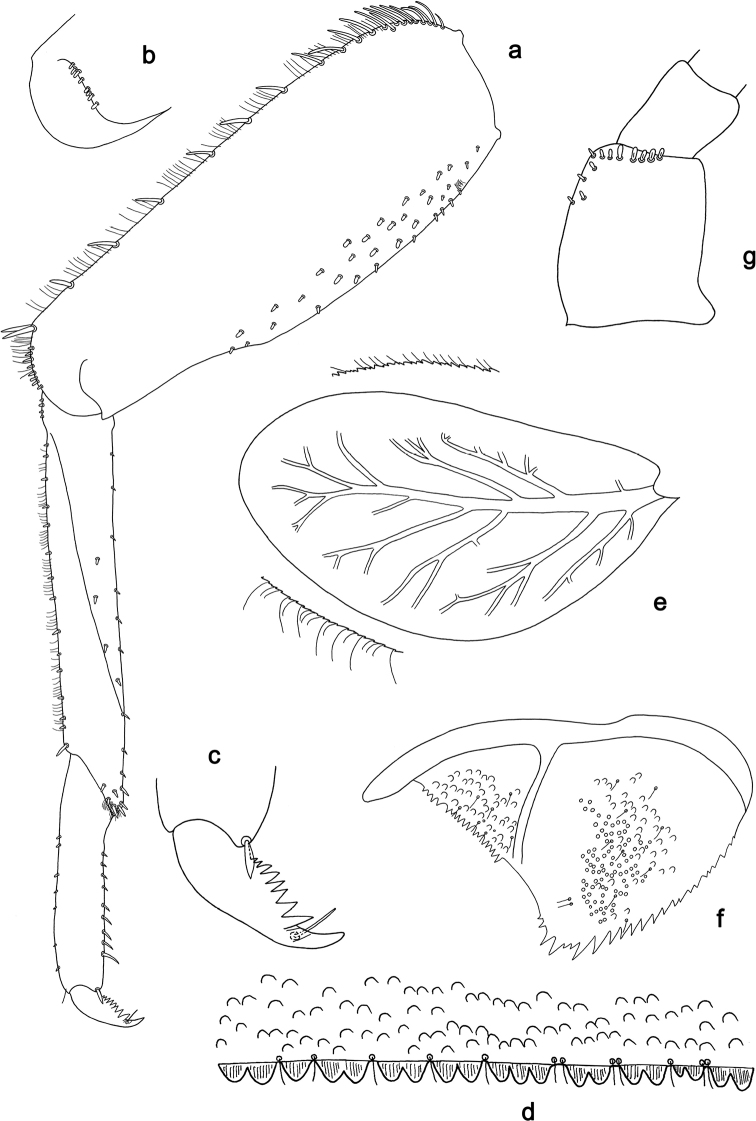
*Branchiobaetisminangkabau* sp. nov., larva **a** foreleg **b** fore femur apex, posterior view **c** fore claw **d** tergum IV **e** tergalius IV **f** paraproct **g** base of antenna.

Figs 20 , 21 , 25a , 26d , 28b

#### Type material.

***Holotype*.** Indonesia • Sumatra, volcano Talamau, River Pularian; 00°02'15"S, 99°59'24"E; 960 m; 01.IV.2014; leg. M. Gueuning; larva on slide; GBIFCH00592524; MZL. ***Paratypes*.** Same data as holotype; larva on slide; GBIFCH00422480; MZL; 18 larvae in alcohol; GBIFCH00406299, GBIFCH00406308, GBIFCH00406398, GBIFCH00406407, GBIFCH00422240, GBIFCH00422245, GBIFCH00422247, GBIFCH00422257, GBIFCH00422262, GBIFCH00422269, GBIFCH00422481, GBIFCH00422527, GBIFCH00422534, GBIFCH00423110, GBIFCH00980904; MZL. Indonesia • West Sumatra, Sawahlunto, Talawi Hilir, Dusun Talimato, UB Farm; 0°35'52"S, 100°43'02"E; 305 m; 25.X.2013; leg. M. Balke; larva on slide; GBIFCH00763628; MZB (temporarily housed in MZL); larva on slide; GBIFCH00592445; MZL; 2 larvae in alcohol; GBIFCH00975608, GBIFCH00980900; MZL.

#### Differential diagnosis.

**Larva.** Following combination of characters distinguish *B.minangkabau* sp. nov. from other species of *Branchiobaetis* gen. nov.: A) labial palp segment II with small protuberance; segment III slightly pentagonal, apically slightly concave, with projecting point (Fig. 20h); B) dorsal margin of femur with row of long, spine-like setae, denser in basal area (Fig. 21a); C) posterior margin of tergites: I smooth, without spines; II–IV with rounded spines, wider than long, partly fused at base; V–IX with triangular spines, narrower and longer towards last segment (Fig. 21d); posterior margin of sternites: I–VI smooth, without spines; VII and VIII with small, spaced, triangular spines; IX with small, triangular spines; D) paraproct without short, stout, apically rounded setae along posterior margin (Fig. 21f).

#### Description.

**Larva** (Figs 20, 21, 25a, 26d). Body length 6.5–8.5 mm. Cerci: ca. 2/3 of body length. Paracercus: ca. 1/2 cerci length. Antenna: ca. 2.5× as long as head length.

***Colouration*** (Fig. 25a). Head, thorax and abdomen dorsally brown, abdominal segments I and X lighter, abdomen laterally on segments II–IX whitish; head, thorax and abdomen ventrally light brown. Legs with dark brown coxae, femur light brown, apically and dorsally along margin dark brown, with large, medial, dark brown spot; tibia light brown; tarsus basally light brown, dark brown in distal half. Caudalii light brown, primary swimming setae dark brown.

***Precursors of turbinate eyes*** (Fig. 25e) in male last instar larvae representing a pair of egg-shaped maculae; in the middle of this macula, a smaller, round, elevated area with well-expressed facets, ca. eight facets in diameter; peripheral area of the macula with indistinct facets.

***Antenna*** (Fig. 21g). Scape distally and outside distolaterally with short, stout, apically rounded setae.

***Labrum*** (Fig. 20a). Length 0.6× maximum width. Submarginal arc of setae composed of 11–13 long, simple setae.

***Right mandible*** (Fig. 20b, c). Incisor and kinetodontium distally cleft. Incisor blade-like with three denticles; kinetodontium with four denticles. Minute setae outside laterally on first denticle (present on fresh mandibles only). Margin between prostheca and mola straight, with minute denticles.

***Left mandible*** (Fig. 20d, e). Incisor blade-like with three denticles; kinetodontium with three denticles. Minute setae outside laterally on first denticle (present on fresh mandibles only). Margin between prostheca and mola straight, with minute denticles towards subtriangular process.

Both mandibles with lateral margins almost straight.

***Hypopharynx and superlinguae*** (Fig. 20f). Lingua as long as superlinguae. Lingua longer than broad; medial tuft of stout setae well developed, long. Superlinguae distally rounded; lateral margins rounded; fine, long, simple setae along distal margin.

***Maxilla*** (Fig. 20g). Galea-lacinia ventrally with one simple, apical seta under canines. Medially with one pectinate, spine-like seta and three or four medium, simple setae. Maxillary palp as long as galea-lacinia; palp segment II 1.1× as long as segment I; setae on maxillary palp fine, simple, scattered over surface of segments I and II.

***Labium*** (Fig. 20h). Inner margin of glossa with approx. eight spine-like setae, increasing in length distally; apex with two long and one medium, robust setae; outer margin with two or three spine-like setae; Paraglossa with one simple seta in posterolateral area; dorsally with two long, spine-like setae near inner margin. Labial palp with segment I approx. as long as length of segments II and III combined. Segment I ventrally with short, fine, simple setae. Segment II with small, distomedial protuberance; distomedial protuberance 0.3× width of base of segment III; ventral surface with short, fine, simple setae; dorsally with 6–9 spine-like setae near outer margin, not always in a row. Segment III slightly pentagonal, apically slightly concave, with projecting point; length approx. maximum width; ventrally covered with short, spine-like, simple setae and short, fine, simple setae.

***Foreleg*** (Fig. 21a–c). Ratio of foreleg segments 1.4:1.0:0.6:0.2. ***Femur***. Length ca. 3× maximum width. Dorsal margin with row of 15–20 long, curved, spine-like setae, basally denser. Many short, stout, apically rounded setae scattered along ventral margin. Apex rounded, with pair of long, spine-like setae and some short, stout setae. ***Tibia***. Dorsal margin with row of short, stout setae. On surface few such setae along patella-tibial suture. Ventral margin with row of short, curved, spine-like setae, on apex a tuft of fine, simple setae. ***Tarsus***. Dorsal margin with row of short, stout setae. ***Claw*** with one row of eight or nine denticles.

***Terga*** (Fig. 21d). Surface with irregular rows of U-shaped scale bases. Posterior margin of tergites: I smooth, without spines; II–IV with rounded spines, wider than long, partly fused at base; V–IX with triangular spines, narrower and longer towards last segment. Posterior margin of sternites: I–VI smooth, without spines; VII–VIII with small, spaced, triangular spines; IX with small triangular spines.

***Tergalii*** (Figs 21e, 26d). Tracheae not reaching inner and outer margins; indistinct broad, light brown band along main trunk of tracheae on anal side. Tergalius I as long as 2/3 of segment II, tergalius IV as long as length of segments V and 2/3 VI combined, tergalius VII as long as length of segments VIII and 1/3 IX combined.

***Paraproct*** (Fig. 21f). Posterior margin with 14–24 stout spines. Without short, stout setae near posterior margin. Surface scattered with scale bases, micropores and fine, simple setae.

#### Etymology.

Dedicated to the indigenous Minangkabau people, who live in the area of Sumatra where the specimens were collected.

#### Distribution.

Indonesia: Sumatra (Fig. 28b).

#### Biological aspects.

The specimens were collected on altitudes of 300 m and 960 m, most of them in a stream with the following physical conditions: slope 25%, width of stream 3–20 m, depth ca. 1.5 m, velocity slow in pool and 0.8 m/s in cascade, pH 8, stream bed dominated by bedrock and stones with patches of sand.

### ﻿7. Branchiobaetis jhoanae sp. nov.

Taxon classificationAnimaliaEphemeropteraBaetidae

D253B1D5-004F-578B-B0EF-114DE88E750E

https://zoobank.org/92CD6523-BB67-48E6-BA4E-09B10D3CA416

**Figure 22.**
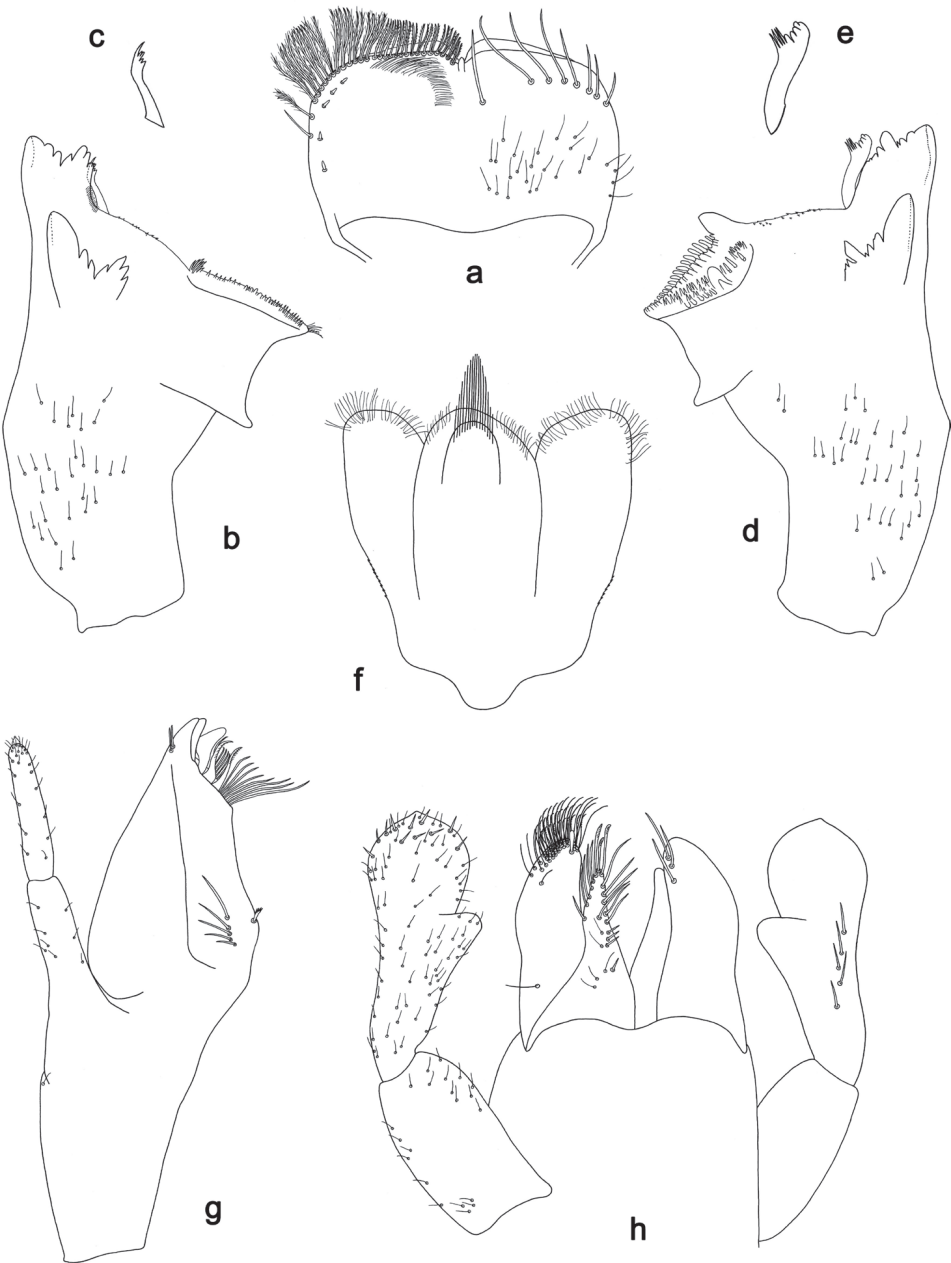
*Branchiobaetisjhoanae* sp. nov., larva **a** labrum (left: ventral view, right: dorsal view) **b** right mandible **c** right prostheca **d** left mandible **e** left prostheca **f** hypopharynx and superlinguae **g** maxilla **h** labium (left: ventral view, right: dorsal view).

**Figure 23.**
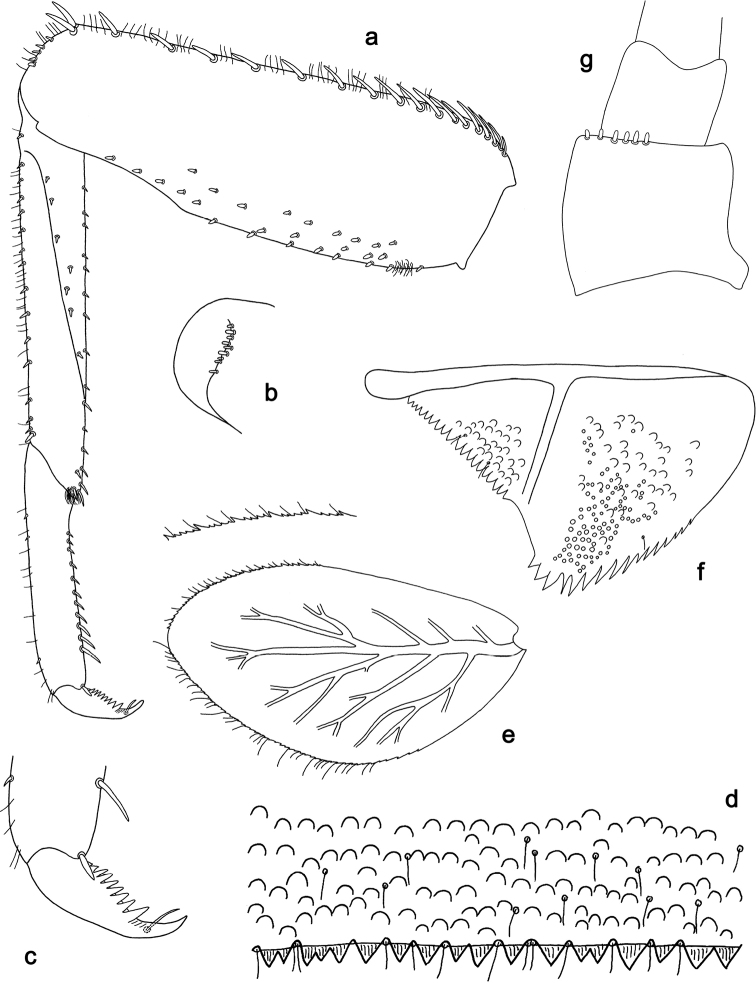
*Branchiobaetisjhoanae* sp. nov., larva **a** foreleg **b** fore femur apex, posterior view **c** fore claw **d** tergum IV **e** tergalius IV **f** paraproct **g** base of antenna.

Figs 22 , 23 , 25b , 26e , 29

#### Type material.

***Holotype*.** Philippines • S. Luzon, Sorsogon, Bulusan, San Roque; 12°44'N, 124°05'E; 290 m; 26. IX. 1996; leg. J. Mendoza; larva on slide; GBIFCH00592344; PNM. ***Paratypes*.** Same data as holotype; 1 larva on slide; GBIFCH00763660; MZL. Philippines • Cebu, Cebu City, Cantipla Uno; 10°20'48"N, 123°51'57"E; 100 m; 11. IX. 1996; leg. J. Mendoza; larva on slide; GBIFCH00592341; AdMU; larva in alcohol; GBIFCH00515474; AdMU • Cebu, Cebu City, Bgy. Tabunan, sitio Cantipla 1; 10°24'56"N, 123°49'02"E; 753 m; 16.XII.1998; leg. Panganthion; larva on slide; GBIFCH00654920; MZL; 2 larvae in alcohol; GBIFCH00515475, GBIFCH00980901; AdMU.

#### Differential diagnosis.

**Larva.** Following combination of characters distinguish *B.jhoanae* sp. nov. from other species of *Branchiobaetis* gen. nov.: A) labial palp segment II with small, rounded protuberance; segment III slightly pentagonal, apically pointed, ca. 0.7× length of segment II, ca. 1.4× as long as width at base, approx. as long as maximal width (Fig. 22h); B) dorsal margin of femur with row of long, spine-like setae (Fig. 23a); C) posterior margin of tergites: I smooth, without spines; II–IX with triangular spines (Fig. 23d); posterior margin of sternites: I–VI smooth, without spines; VII–IX with small, spaced, triangular spines; D) paraproct without short, stout, apically rounded setae along posterior margin (Fig. 23f).

#### Description.

**Larva** (Figs 22, 23, 25b, 26e). Body length 5.8–7.0 mm. Cerci: ca. ½ of body length. Paracercus: ca. 2/3 of cerci length. Antenna: ca. 2.5× as long as head length.

***Colouration*** (Fig. 25b). Head, thorax, and abdomen dorsally brown; head, thorax and abdomen ventrally light brown to brown. Legs light brown to brown, large brown areas along dorsal margin, apex and on medial surface of femur. Caudalii light brown, primary swimming setae dark brown.

**Figure 24.**
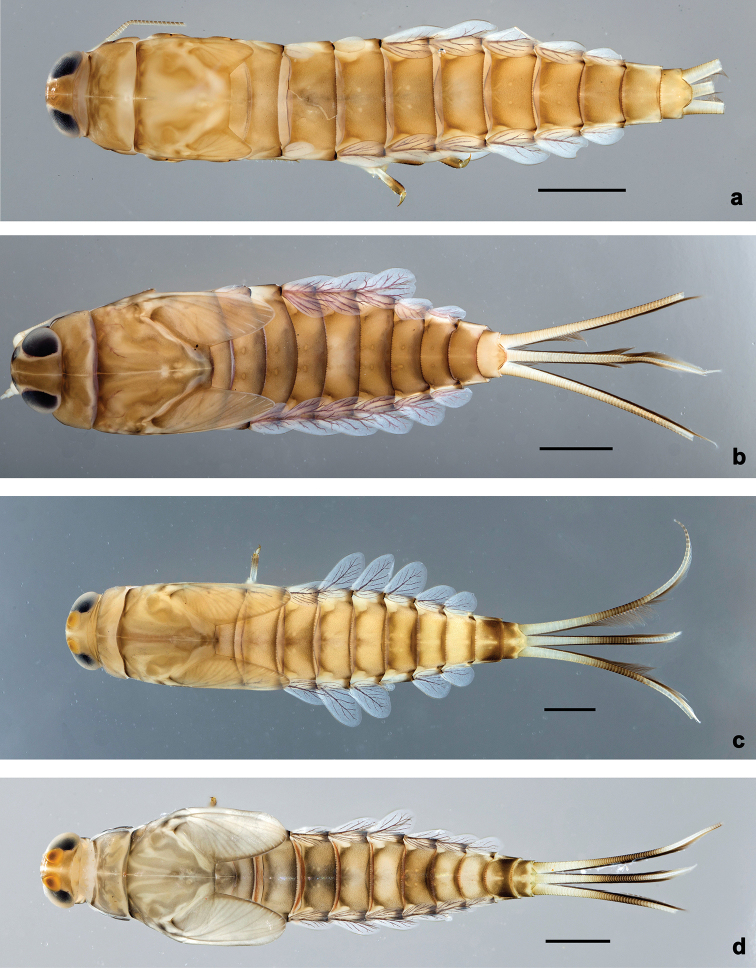
Habitus, larvae, dorsal view **a***Branchiobaetisaduncus* sp. nov. **b***Branchiobaetishamatus* sp. nov. **c***Branchiobaetisjoachimi* sp. nov. (Sumatra, volcano Sago) **d***Branchiobaetisjoachimi* sp. nov. (Sumatra, Bukit Barisan, above Padang).

***Antenna*** (Fig. 23g). Scape distally with short, stout, apically rounded setae.

***Labrum*** (Fig. 22a). Length 0.6× maximum width. Submarginal arc of setae composed of 7–9 long, simple setae.

***Right mandible*** (Fig. 22b, c). Incisor blade-like with three denticles; kinetodontium with four denticles. Margin between prostheca and mola straight, with minute denticles.

**Figure 25.**
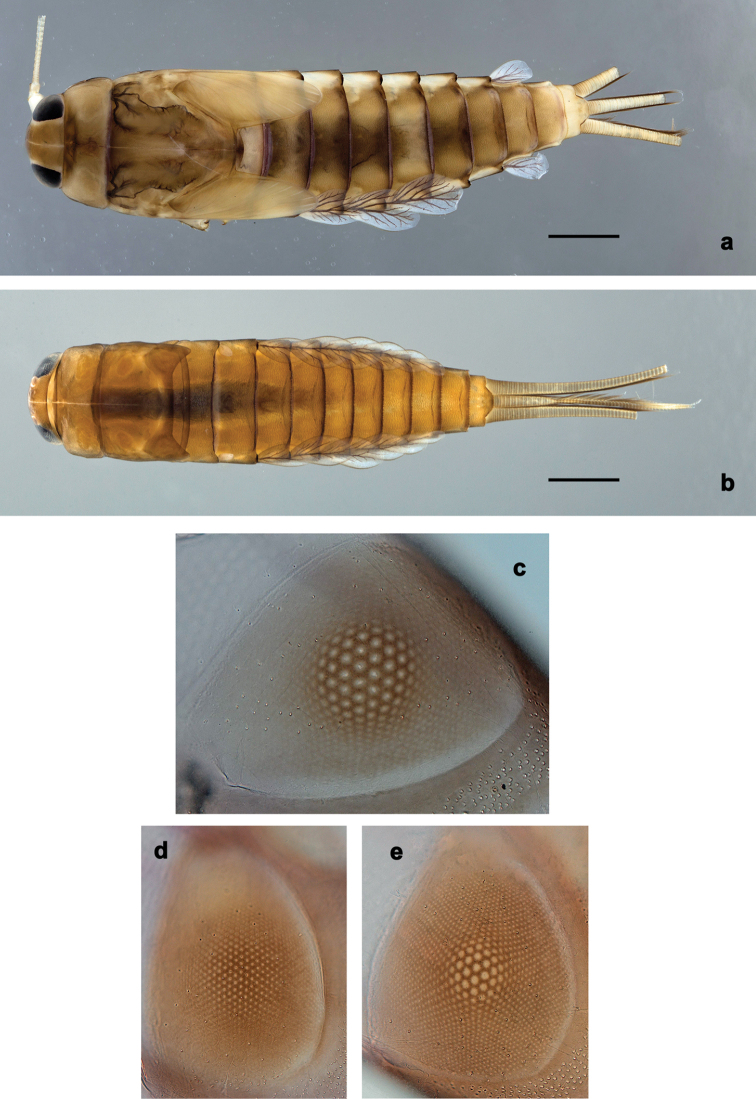
Habitus, larvae, dorsal view **a***Branchiobaetisminangkabau* sp. nov. **b***Branchiobaetisjhoanae* sp. nov. Precursors of turbinate eyes developing in male last instar larvae **c***Branchiobaetishamatus* sp. nov. **d***Branchiobaetisjoachimi* sp. nov. **e***Branchiobaetisminangkabau* sp. nov.

***Left mandible*** (Fig. 22d, e). Incisor blade-like with four denticles; kinetodontium with three denticles. Margin between prostheca and mola straight, with minute denticles towards subtriangular process.

Both mandibles with lateral margins slightly convex.

***Hypopharynx and superlinguae*** (Fig. 22f). Lingua as long as superlinguae. Lingua longer than broad; medial tuft of stout setae well developed, long. Superlinguae distally rounded; lateral margins rounded; fine, long, simple setae along distal margin.

**Figure 26.**
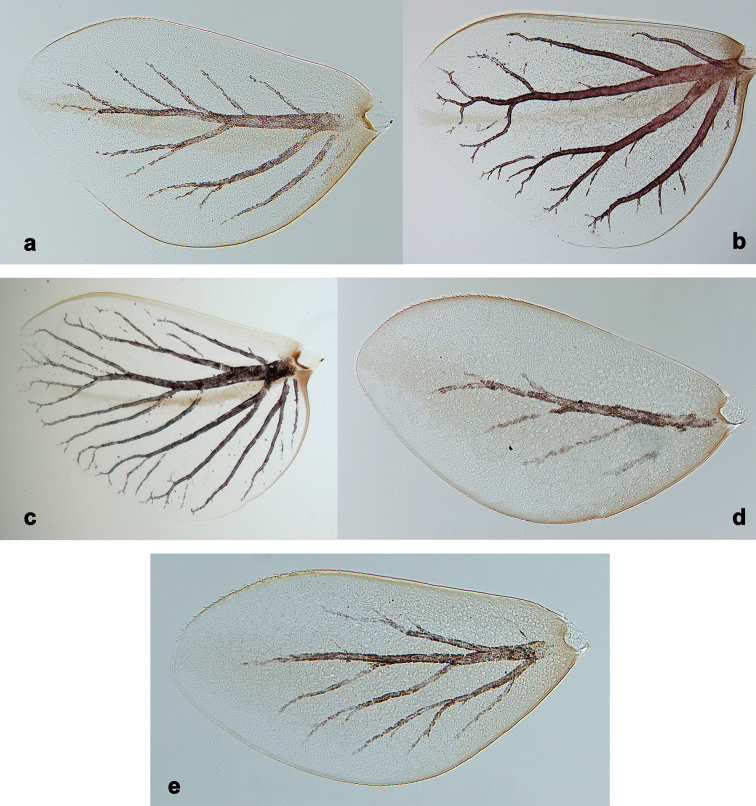
Larva, tergalii IV **a***Branchiobaetisaduncus* sp. nov. **b***Branchiobaetishamatus* sp. nov. **c***Branchiobaetisjoachimi* sp. nov. **d***Branchiobaetisminangkabau* sp. nov. **e***Branchiobaetisjhoanae* sp. nov.

***Maxilla*** (Fig. 22g). Galea-lacinia ventrally with two simple, apical seta under canines. Medially with one pectinate, spine-like seta and 4–6 medium, simple setae. Maxillary palp approx. as long as galea-lacinia; palp segment II approx. as long as segment I; setae on maxillary palp fine, simple, scattered over surface of segments I and II.

**Figure 27.**
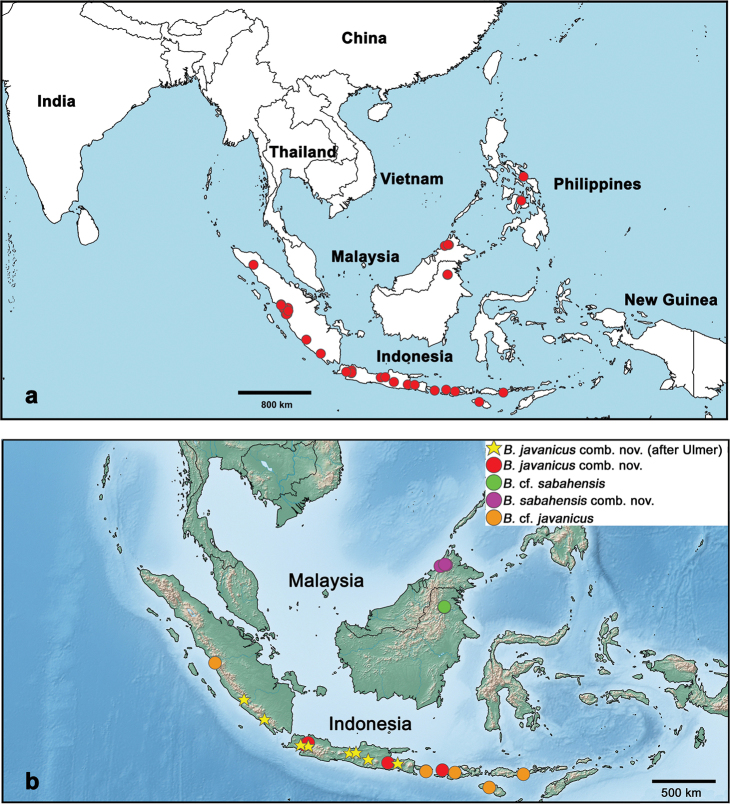
Distribution maps **a***Branchiobaetis* gen. nov. in Southeast Asia **b** Known species of *Branchiobaetis* gen. nov.

***Labium*** (Fig. 22h). Inner margin of glossa with eight or nine spine-like setae, increasing in length distally; apex with two long and one medium, robust, pectinate setae; outer margin with approx. five spine-like setae; Paraglossa with one simple seta in anterolateral area and one simple seta in posterolateral area; dorsally with three long, spine-like setae near inner margin. Labial palp with segment I 0.7× as long as length of segments II and III combined. Segment I ventrally with short, fine, simple setae. Segment II with small, rounded, distomedial protuberance; distomedial protuberance 0.3× width of base of segment III; ventral surface with short, fine, simple setae; dorsally with five or six spine-like setae near outer margin. Segment III slightly pentagonal, apically pointed; length approx. maximum width; ventrally covered with short, spine-like, simple setae and short, fine, simple setae.

**Figure 28.**
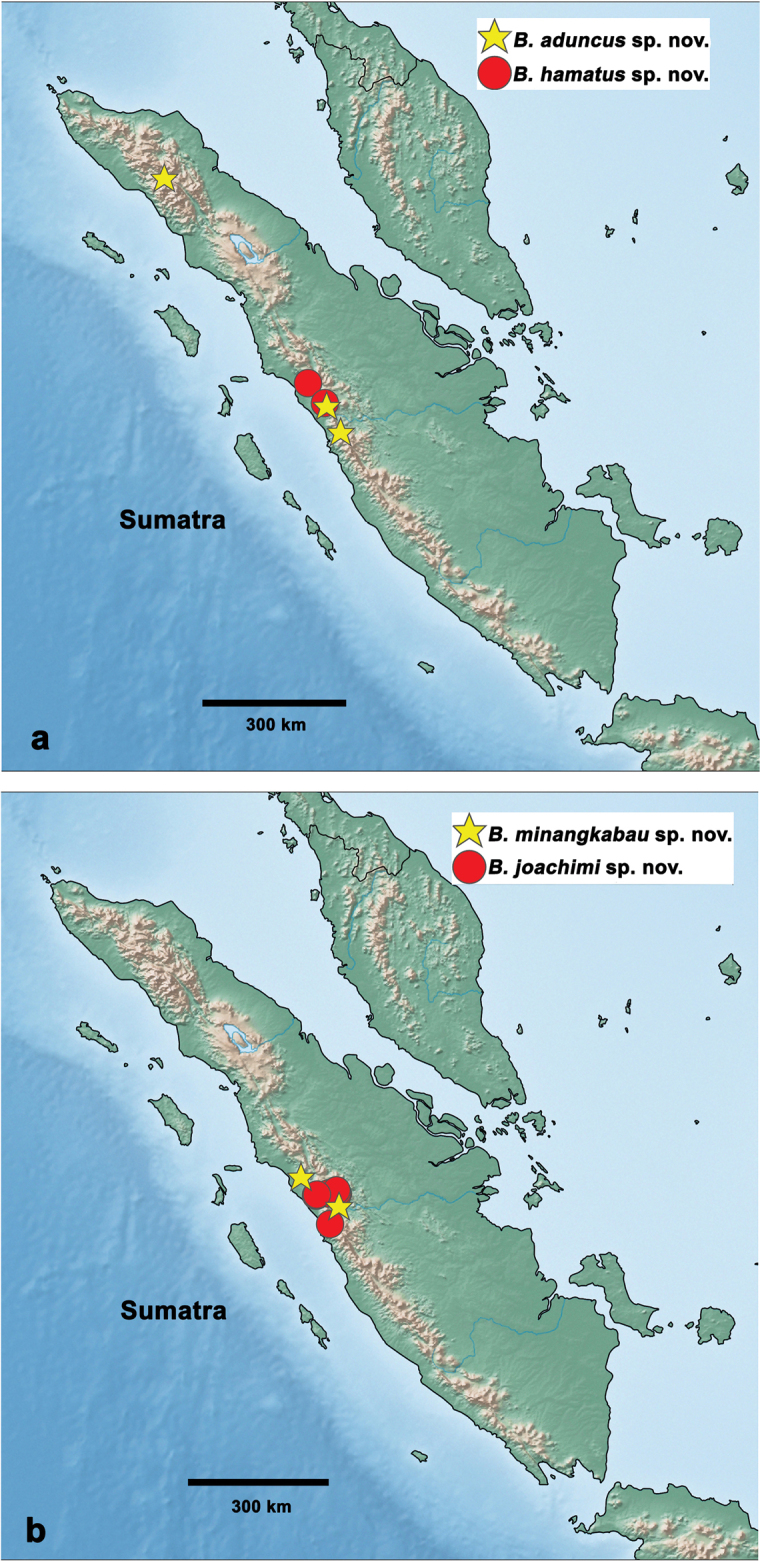
Distribution maps **a, b** new species of *Branchiobaetis* gen. nov. in Sumatra.

***Foreleg*** (Fig. 23a–c). Ratio of foreleg segments 1.3:1.0:0.6:0.2. ***Femur***. Length ca. 3× maximum width. Dorsal margin with row of 14–21 long, curved, spine-like setae, basally denser. Many short, stout setae scattered along ventral margin. Apex rounded, with pair of long, spine-like setae and some short, stout setae. ***Tibia***. Dorsal margin with row of short, stout setae. On surface few such setae along patella-tibial suture. Ventral margin with row of short, curved, spine-like setae, on apex a tuft of fine, simple setae. ***Tarsus***. Dorsal margin with row of short, stout setae and fine, simple setae. ***Claw*** with one row of nine or ten denticles.

***Terga*** (Fig. 23d). Surface with irregular rows of U-shaped scale bases and fine simple setae. Posterior margin of tergites: I smooth, without spines; II–IX with triangular spines. Posterior margin of sternites: I–VI smooth, without spines; VII–IX with small, spaced, triangular spines.

***Tergalii*** (Figs 23e, 26e). Tracheae extending to inner and outer margins; indistinct, broad, light brown band along main trunk of tracheae on anal side. Tergalius I as long as 1/2 of segment II, tergalius IV as long as length of segments V and 1/4 VI combined, tergalius VII as long as length of segments VIII and 1/4 IX combined.

***Paraproct*** (Fig. 23f). Posterior margin with 14–18 stout spines. Without short, stout setae near posterior margin. Surface scattered with scale bases and micropores.

#### Etymology.

Dedicated to Dr. Jhoana M. Garces (Philippines) for her great contribution to the knowledge of mayflies from the Philippines.

#### Distribution.

**Figure 29.**
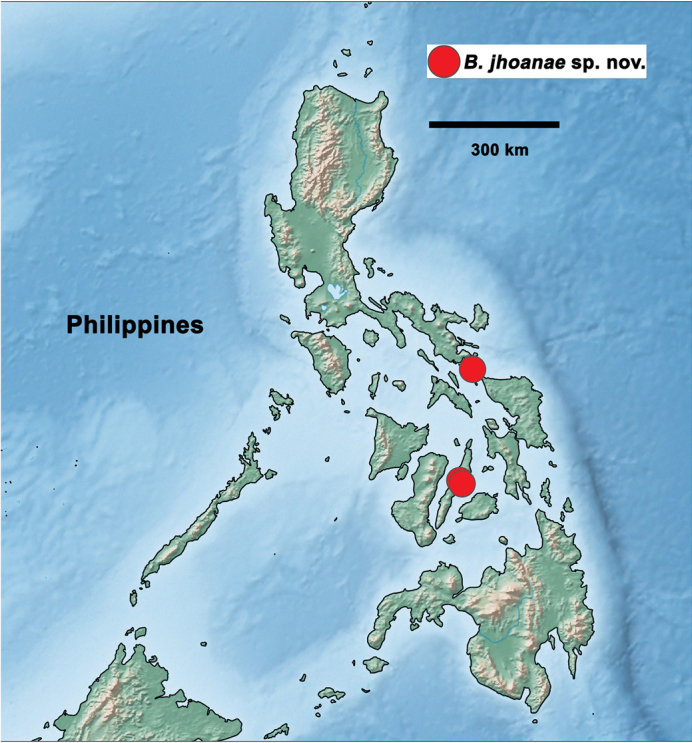
Distribution map. *Branchiobaetisjhoanae* sp. nov. in the Philippines.

Philippines: Luzon, Cebu (Fig. 29).

#### Biological aspects.

The specimens were collected on altitudes between 100 m and 750 m, on Cebu in stream runs on bottom gravel or rock surface.

### ﻿Key to the species of *Branchiobaetis* gen. nov. (larvae)

**Table:** 

1	Dorsal margin of femur with row of medium, spine-like setae and many short, apically rounded setae in two or three irregular rows along margin; short, stout, apically rounded setae in middle area of anterior surface of femur (Fig. 19a)	***B.joachimi* sp. nov.**
–	Dorsal margin of femur with row of medium to long, spine-like setae, no additional row of short, apically rounded setae, or one single row of short, hooked setae along margin; no stout setae in middle area of anterior surface of femur (Figs 15a, 21a)	**2**
2	Many short, stout, hook-like setae along dorsal margin of femur, tibia and tarsus (Fig. 15a, c); labial palp segment III apically rounded (Fig. 14j)	**3**
–	No short, hook-like setae along dorsal margin of femur, tibia or tarsus (Fig. 21a); labial palp segment III apically pointed (Fig. 22h)	**4**
3	Posterior margin of tergite IV with apically rounded spines (Fig. 15f); tergalius IV with convex apex (Fig. 15g)	***B.aduncus* sp. nov.**
–	Posterior margin of tergite IV with triangular, pointed spines (Fig. 17e); tergalius IV apically with slight concavity (Fig. 17f)	***B.hamatus* sp. nov.**
4	Labial palp segment III distally wide, with projecting point, apical margin slightly concave (Fig. 20h); posterior margin of tergite IV with rounded spines (Fig. 21d)	***B.minangkabau* sp. nov.**
–	Labial palp segment III distally pointed, point not projecting, apical margin not concave (Fig. 22h); posterior margin of tergite IV with triangular, pointed spines	**5**
5	Incisor of right mandible with ventral denticle; labial palp segment III rather short, ca. 0.5× length of segment II (Fig. 12d; Müller-Liebenau 1984b: fig. 3b, e); Borneo	***B.sabahensis* comb. nov.**
–	Incisor of right mandible without ventral denticle; labial palp segment III rather long, ca. 0.7× length of segment II (Figs 6b, 22b, h; Müller-Liebenau 1981: fig. 1b, e)	**6**
6	Posterior margin of tergite IV with triangular spines, wider than long; tergalius IV rather oblong; paraproct without stout setae along margin (Fig. 23d–f); Philippines	***B.jhoanae* sp. nov.**
–	Posterior margin of tergite IV with triangular spines, longer than wide; tergalius IV with bellied shape; paraproct with stout, apically rounded setae along margin (Figs 5e, 7j; Müller-Liebenau 1981: fig. 1m, pl. 1.1); Indonesia (Sunda Islands)	***B.javanicus* comb. nov.**

### ﻿Genetics

The interspecific genetic distances between the species of *Branchiobaetis* gen. nov. are rather high, between 13% and 21% (Table 3).

**Table 3.** Intraspecific (bold) and interspecific genetic distances of *Branchiobaetis* gen. nov. species (COI; Kimura 2-parameter); green lines indicate species delimitation hypothesis according to the ASAP method.

		1	2	3	4	5	6	7	8	9	10	11	12	13	14	15	16	17
**1**	B.cf.javanicus (Sumbawa)																	
**2**	B.cf.javanicus (Sumbawa)	**0.00**																
**3**	B.cf.javanicus (Bali)	0.12	0.12															
**4**	B.cf.javanicus (Sumatra)	0.21	0.21	0.18														
**5**	B.cf.javanicus (Sumatra)	0.21	0.21	0.18	**0.00**													
**6**	*B.aduncus* sp. nov.	0.16	0.16	0.19	0.21	0.21												
**7**	*B.hamatus* sp. nov.	0.19	0.19	0.20	0.18	0.18	0.19											
**8**	*B.hamatus* sp. nov.	0.19	0.19	0.20	0.18	0.18	0.19	**0.00**										
**9**	*B.hamatus* sp. nov.	0.19	0.19	0.20	0.18	0.18	0.19	**0.00**	**0.00**									
**10**	*B.joachimi* sp. nov.	0.20	0.20	0.19	0.20	0.20	0.19	0.20	0.20	0.20								
**11**	*B.joachimi* sp. nov.	0.19	0.19	0.18	0.20	0.20	0.20	0.19	0.19	0.19	**0.01**							
**12**	*B.joachimi* sp. nov.	0.20	0.20	0.18	0.20	0.20	0.20	0.20	0.20	0.20	**0.01**	**0.00**						
**13**	*B.joachimi* sp. nov.	0.20	0.20	0.19	0.20	0.20	0.19	0.20	0.20	0.20	**0.00**	**0.01**	**0.01**					
**14**	*B.joachimi* sp. nov.	0.19	0.19	0.19	0.21	0.21	0.19	0.19	0.19	0.19	**0.05**	**0.05**	**0.05**	**0.05**				
**15**	*B.minangkabau* sp. nov.	0.15	0.15	0.17	0.13	0.13	0.17	0.19	0.19	0.19	0.20	0.20	0.20	0.20	0.20			
**16**	*B.minangkabau* sp. nov.	0.16	0.16	0.17	0.13	0.13	0.17	0.19	0.19	0.19	0.20	0.20	0.20	0.20	0.20	**0.00**		
**17**	*B.minangkabau* sp. nov.	0.15	0.15	0.17	0.13	0.13	0.17	0.19	0.19	0.19	0.20	0.20	0.20	0.20	0.20	**0.00**	**0.00**	
**18**	*B.jhoanae* sp. nov.	0.20	0.20	0.20	0.18	0.18	0.19	0.19	0.19	0.19	0.17	0.17	0.17	0.17	0.18	0.19	0.19	0.19

## ﻿Discussion

### ﻿Relationship, characters, and affinities of *Branchiobaetis* gen. nov.

The new genus *Branchiobaetis* gen. nov. obviously belongs to the family Baetidae, based on the turban eyes of the male imago (Fig. 9b), the forewing with intercalary veins (Fig. 9c), the diminished, narrowed hind wings with strongly reduced venation of male and female imago (Fig. 9e) as well as a series of larval characters, e.g. Y-shaped frontal suture ventral of lateral ocelli, labrum with distinctly expressed medial incision (Fig. 12b), kinetodontium fused with mandible and with incisor (Fig. 6a, b), left prostheca stout stick-like, apically denticulate (Fig. 6a), femur with apical anterior outer projection curved toward inner side of femur (Fig. 15a) (Kluge 2004; Kluge and Novikova 2011). Based on the rank-free system of Kluge (Kluge 2004; Kluge and Novikova 2011), *Branchiobaetis* gen. nov. belongs to the Anteropatellata, because a patella-tibial suture is present on all legs of larva, female subimago and female imago, including forelegs (Figs 7a–c, 8a); to Baetovectata because of the forewings with double intercalaries (Fig. 9a, c) and the 2^nd^ segment of the subimaginal gonostylus developing under larval cuticle bent medially (Fig. 10b); and to Baetungulata or Baetinae (sensu Kazlauskas 1972) because of the claws with one row of denticles on inner-anterior side and a maxillary palp with two segments (Figs 7k, 14i) (Kluge and Novikova 2011). Finally, the new genus is part of the Baetofemorata or the “*Baetis* complex” sensu Waltz and McCafferty (1997), because each larval leg has a femoral patch and subimagines of both sexes have all tarsomeres covered with blunt microlepids (Fig. 8c, d, h) (Kluge and Novikova 2011).

Most interesting in the characters of *Branchiobaetis* gen. nov. is the presence of accessory gills in all species, one finger-like pair ventrally between fore coxa and prosternum and one gill on each maxilla outside between stipes and cardo (Figs 1a, b, 16j; Müller-Liebenau 1984b: fig. 3i). These gills are connected to the tracheal system and have tracheae inside, their respiratory function is therefore probable. However, their size is small in relation to the body size and the size of the tergalii, which are large and with many tracheae. It remains unclear if their contribution to respiration is substantial or rather negligible. In addition, some of the species are reported to live in fast flowing and cold water, where we can expect a high oxygen content and therefore a less importance of gills. Accessory gills are rare in Baetidae and in Ephemeroptera in general, an overview and phylogenetic discussion is given by Staniczek (2010) and Zhou (2010). Mostly, these accessory gills are associated with coxae or thoracic sterna, or with maxillae, similar to *Branchiobaetis* gen. nov.; a multiple convergent development of these accessory gills is assumed (Staniczek 2010; Zhou 2010). In Baetidae, at least three different types of accessory gills were reported: coxal gills (located between coxae and sterna, or between coxae and trochanter), maxillary gills located between stipes and cardo and maxillary gills located at the maxillary palp (Müller-Liebenau 1984b; Gattolliat and Sartori 1999; Dominguez et al. 2006; Staniczek 2010; Zhou 2010; Gattolliat 2012; Shi and Tong 2015; Kluge and Bernal Vega 2018). *Moribaetis* Waltz & McCafferty, 1985 has very similar accessory coxal gills compared to *Branchiobaetis* gen. nov. (Kluge and Bernal Vega 2018: figs 85, 86). This is probably convergent, as other characters are very different and *Moribaetis* belongs to Baetungulata-non-Baetofemorata or the “non-Baetis complex” of Baetinae (sensu Waltz et al. 1994; Waltz and McCafferty 1997) (no femoral patch), whereas *Branchiobaetis* gen. nov. is part of Baetofemorata (Waltz and McCafferty 1985; Kluge and Novikova 2014; Kluge and Bernal Vega 2018).

RhodobaetisJacob, 2003, subgenus of Baetis, is characterized by peculiar, stout, apically rounded setae, generally called spatulae, on the antennal scape and pedicel, which usually appear as well on abdominal terga (Müller-Liebenau 1984a: figs 1f, 34; Jacob 2003; Godunko et al. 2004; Soldán et al. 2005; Soldán and Godunko 2006; Gattolliat et al. 2018; Yanai et al. 2018: figs 12A, 13C; Kluge 2022). The same type of setae is always present on antennal scapes of *Branchiobaetis* gen. nov., but only exceptionally one or two on pedicels and they never appear on abdominal terga. However, important differences between both groups are a developed, slender sterno-styliger muscle in *Branchiobaetis* gen. nov. (Fig. 10f; absent in *Rhodobaetis*), accessory coxal and maxillary gills in *Branchiobaetis* gen. nov. (Fig. 1a, b; usually absent in *Rhodobaetis*; Kluge 2022), and the folding of the gonostyli developing under cuticle of last instar male larvae (“*Branchiobaetis*-type” (see below) vs. “*Baetis*-type” in *Rhodobaetis*; Fig. 4a–d; Kluge 2004: fig. 29H). In specimens of Baetis (Rhodobaetis) illiesi Müller-Liebenau, 1984, from Vietnam, we discovered bubble-like membranous swellings on the legs similar to *Branchiobaetis* gen. nov. and auxiliary gills at base of forecoxae, but no maxillary gills. This is exceptional for *Rhodobaetis* and assumed to be convergent. The folding of the gonostyli developing under the cuticle of a last instar male larva of *B.illiesi* from Vietnam was in the “*Baetis*-type” (authors, unpublished observation).

There are also some similarities between *Branchiobaetis* gen. nov. and *Philibaetis* Kaltenbach & Gattolliat, 2021, from the Philippines: labrum shape and dorsal, submarginal arc of setae; blade-like incisors of both mandibles; maxillary palp with pointed apex; paraglossae laterally slightly rolling, apex truncate and slightly bent inwards (Kaltenbach et al. 2021). However, these similarities are probably due to convergence; *Branchiobaetis* gen. nov. is part of Baetofemorata (presence of femoral patch) and *Philibaetis* belongs to Baetungulata-non-Baetofemorata (absence of femoral patch; Kaltenbach et al. 2021). There are also other important differences: e.g., *Branchiobaetis* gen. nov. with accessory gills (missing in *Philibaetis*); labrum medioventrally without additional, submarginal row of lanceolate setae (present in *Philibaetis*); right prostheca distolaterally denticulate (not apically denticulate as in *Philibaetis*); labial palp segment II with protuberance (missing in *Philibaetis*); scape with stout, apically rounded setae (missing in *Philibaetis*); folding of developing gonostyli under larval cuticle in “*Branchiobaetis*-type” (see below; *Philibaetis* similar to “*Labiobaetis*-type”) (Figs 1a, b, 4a–d, 14a, j, 15i; Kaltenbach et al. 2021: figs 1b, e, f, l, 2d, 3d, 10a, b).

### ﻿Membranous swellings on the legs of *Branchiobaetis* gen. nov.

All species of *Branchiobaetis* gen. nov. have particular, membranous, bubble-like structures at the legs of the larvae. They were never described in Baetidae: a swelling of the articulatory membrane between coxa and trochanter of all legs and a swelling of the articulatory membrane between coxa and pleurite of forelegs and middle legs (less developed) (Figs 1b, 2a–c). The degree of development of these swellings seems to vary between individuals. There are no tracheae inside and no other special structure, it seems to be simply convex, enlarged membranes. The function of these structures remains unclear, we do not consider them to be accessory gills. One possible explanation is that these structures support respiration through the membranous parts of the integument by increasing the membranous surface of the body. Oxygen and CO_2_ exchange through the membranous parts of the integument is known from many aquatic insects (Eidmann and Kühlhorn 1970). The involvement of air compartments inside the body of aquatic insects in their hydrostatic balance was also discussed (Eidmann and Kühlhorn 1970) and could be another possible function. However, all possible explanations of the function of the bubble-like membrane swellings at the legs of *Branchiobaetis* gen. nov. remain speculative without further investigation.

### ﻿Subimaginal gonostyli developing under larval cuticle

In *Branchiobaetis* gen. nov., the second segment of the subimaginal gonostylus developing under the cuticle of last instar male larvae is bent medially as in other Baetofemorata. However, the 3^rd^ segment is sharply bent laterally, which is peculiar for this genus (“*Branchiobaetis*-type” of folding) and different from the “*Baetis*-type” of folding (Fig. 4a–d; Kluge 2004: fig. 29H). Other types of folding in Baetidae are illustrated in Kluge (2004: fig. 29E–J).

### ﻿Genetics

The interspecific genetic distances of *Branchiobaetis* gen. nov. are in line with values reported for other Baetidae in Southeast Asia (Labiobaetis: 11–24% in Indonesia, 15–27% in the Philippines; Kaltenbach and Gattolliat 2019; Kaltenbach et al. 2020a). Ball et al. (2005) reported a mean interspecific, congeneric distance of 18% for mayflies from the United States and Canada. The intraspecific distances are very low in most cases as expected, ranging from 0% to 1% (K2P). This result is certainly biased as it is based on a limited number of sequenced specimens per species, which were partly from a single population. The exception is *B.joachimi* sp. nov., where one specimen of a more distant location has a genetic distance of 5% (K2P) to all three other specimens. This larger genetic distance may be explained by a possible isolation of the location causing a higher distance. Intraspecific distances of 4–6% were also reported in some cases for *Labiobaetis* species in New Guinea, Indonesia, Borneo, and the Philippines (Kaltenbach and Gattolliat 2018, 2019, 2020; Kaltenbach et al. 2020a), as well as in aquatic beetles in the Philippines (Komarek and Freitag 2020). Ball et al. (2005) also reported a case with 6% intraspecific distance in a mayfly in North America and intraspecific K2P distances of more than 3.5% are not uncommon within Plecoptera as well (Gill et al. 2015; Gattolliat et al. 2016).

For *B.javanicus* comb. nov., we do not have a COI sequence from Java, where the type locality is. However, we have sequences from larvae with the same morphology as *B.javanicus* comb. nov. from Sumbawa, Bali and Sumatra. The specimens from these three locations present important genetic distances to each other (12–21%; K2P; Table 3). According to the most likely scenario of hypothetical species obtained with the ASAP method (Table 3), the specimens of B.cf.javanicus comb. nov. from Sumbawa and Bali are retained as one hypothetical species and the specimens from Sumatra as another one. However, the second likely scenario obtained with the ASAP method also separated the specimens from Bali and Sumbawa as different hypothetical species. This is also supported by the ML reconstruction (Suppl. material 1). We are treating them all as B.cf.javanicus comb. nov. for now. It remains unclear, if it is a question of cryptic diversity, different mitochondrial lineages of the same species or something else (see also the discussion of Molecular Operational Taxonomic Units (MOTUs) in Kaltenbach et al 2020a: table 4). Additional material and investigations will be necessary to confirm their status. All described new species of *Branchiobaetis* gen. nov. are supported by the species delimitation with the ASAP method (Table 3) and the ML reconstruction (Suppl. material 1).

### ﻿Distribution

*Branchiobaetis* gen. nov. has a wide distribution across Southeast Asia, so far including Indonesia (Greater and Lesser Sunda Islands, Borneo), Malaysia (Borneo), and the Philippines. Taking into account the generally high diversity in Southeast Asia and the rather poor collection activities in the past, with many still unexplored regions, we have to expect more species and an even larger distribution, including most of continental Southeast Asia.

## Supplementary Material

XML Treatment for Branchiobaetis

XML Treatment for Branchiobaetis javanicus

XML Treatment for Branchiobaetis sabahensis

XML Treatment for Branchiobaetis aduncus

XML Treatment for Branchiobaetis hamatus

XML Treatment for Branchiobaetis joachimi

XML Treatment for Branchiobaetis minangkabau

XML Treatment for Branchiobaetis jhoanae

## Supplementary materials

Supplementary material 1 ML reconstruction *Branchiobaetis* gen. nov. Data type image https://binary.pensoft.net/file/780360This dataset is made available under the Open Database License (http://opendatacommons.org/licenses/odbl/1.0/). The Open Database License (ODbL) is a license agreement intended to allow users to freely share, modify, and use this Dataset while maintaining this same freedom for others, provided that the original source and author(s) are credited.Thomas Kaltenbach, Nikita J. Kluge, Jean-Luc Gattolliat
