# Supplementary figures and images for: ﻿A widespread new genus of Baetidae (Baetidae, Ephemeroptera) from Southeast Asia
Source: Zookeys. 2022 Dec 12;1135:1–59. doi: 10.3897/zookeys.1135.93800 (PMC9836713; doi:10.3897/zookeys.1135.93800)

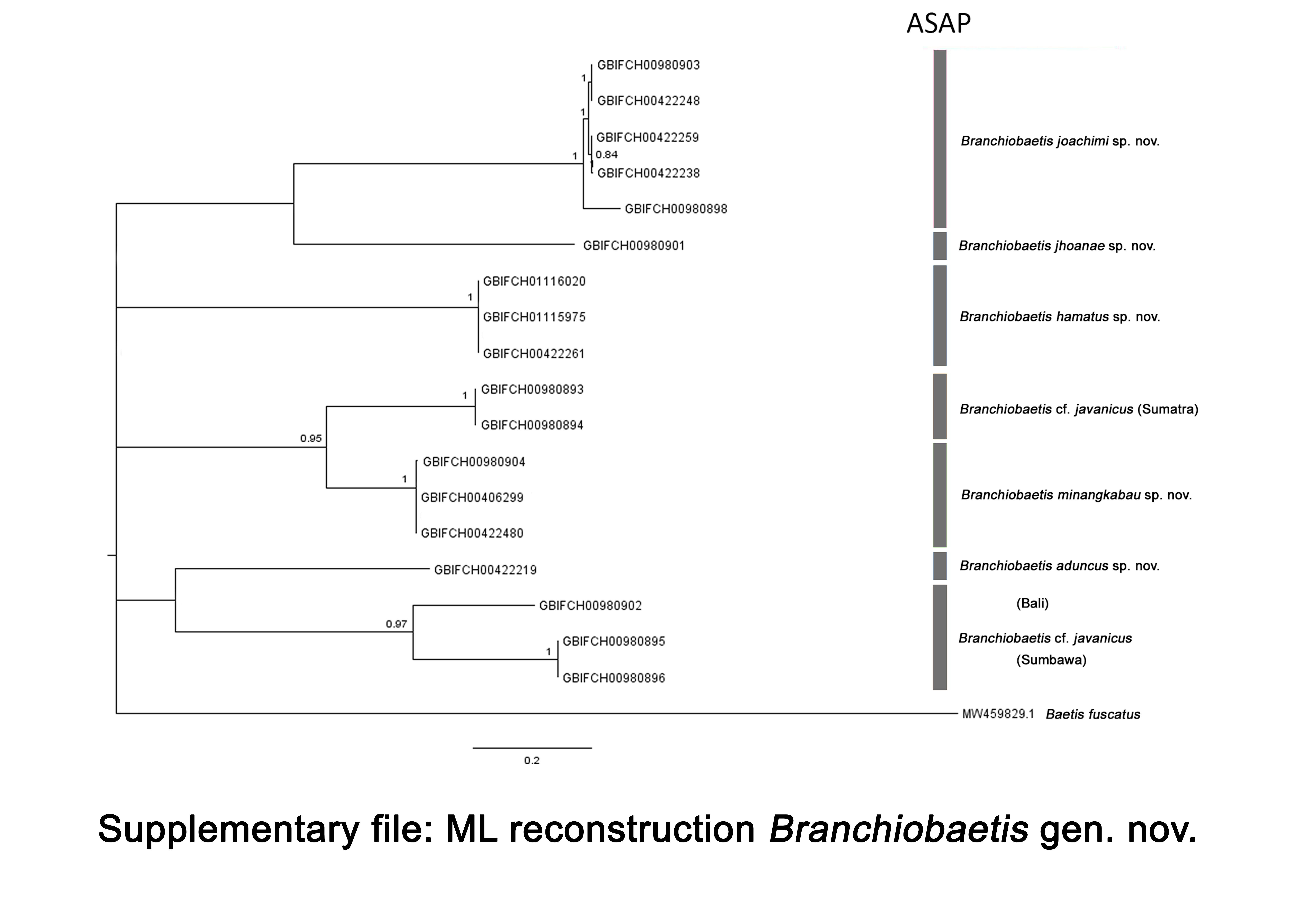

Supplement: Supplementary material 1 — ML reconstruction Branchiobaetis gen. nov. [file zookeys-1135-001_article-93800__-s001.jpg]
